# Intra-pituitary follicle-stimulating hormone signaling regulates hepatic lipid metabolism in mice

**DOI:** 10.1038/s41467-023-36681-z

**Published:** 2023-02-25

**Authors:** Sen Qiao, Samer Alasmi, Amanda Wyatt, Philipp Wartenberg, Hongmei Wang, Michael Candlish, Debajyoti Das, Mari Aoki, Ramona Grünewald, Ziyue Zhou, Qinghai Tian, Qiang Yu, Viktoria Götz, Anouar Belkacemi, Ahsan Raza, Fabien Ectors, Kathrin Kattler, Gilles Gasparoni, Jörn Walter, Peter Lipp, Patrice Mollard, Daniel J. Bernard, Ersin Karatayli, Senem Ceren Karatayli, Frank Lammert, Ulrich Boehm

**Affiliations:** 1 Experimental Pharmacology, Center for Molecular Signaling (PZMS), Saarland University School of Medicine, Homburg, Germany; 2 Assisted Reproduction Center, Northwest Women’s and Children’s Hospital, Xi’an, China; 3 Department of Pharmacology and Therapeutics, McGill University, Montreal, Quebec Canada; 4 Molecular Cell Biology, Center for Molecular Signaling (PZMS), Saarland University School of Medicine, Homburg, Germany; 5 FARAH Mammalian Transgenics Platform, Liège University, Liège, Belgium; 6 Department of Genetics and Epigenetics, Saarland University, Saarbrücken, Germany; 7 IGF, University of Montpellier, CNRS, INSERM, Montpellier, France; 8 Department of Medicine II, Saarland University Medical Center, Saarland University, Homburg, Germany; 9 Present Address: Institute of Cell Biology and Neuroscience, Buchmann Institute for Molecular Life Sciences, Goethe University Frankfurt, Frankfurt am Main, Germany

**Keywords:** Pituitary gland, Metabolic bone disease, Bone

## Abstract

Inter-organ communication is a major hallmark of health and is often orchestrated by hormones released by the anterior pituitary gland. Pituitary gonadotropes secrete follicle-stimulating hormone (FSH) and luteinizing hormone (LH) to regulate gonadal function and control fertility. Whether FSH and LH also act on organs other than the gonads is debated. Here, we find that gonadotrope depletion in adult female mice triggers profound hypogonadism, obesity, glucose intolerance, fatty liver, and bone loss. The absence of sex steroids precipitates these phenotypes, with the notable exception of fatty liver, which results from ovary-independent actions of FSH. We uncover paracrine FSH action on pituitary corticotropes as a mechanism to restrain the production of corticosterone and prevent hepatic steatosis. Our data demonstrate that functional communication of two distinct hormone-secreting cell populations in the pituitary regulates hepatic lipid metabolism.

## Abstract (web summary)

Gonadotropes in the pituitary secrete follicle-stimulating hormone and luteinizing hormone to control gonadal function and fertility, but whether they exert actions on extra-gonadal organs is not fully understood. Here the authors report that gonadotropes regulate liver steatosis independent of the ovaries in mice.

## Introduction

The anterior pituitary gland is a major regulator of mammalian body homeostasis and orchestrates communication between different organs via the coordinated release of distinct hormones^1^. Different pituitary cell types produce growth hormone, prolactin, thyroid-stimulating hormone, adrenocorticotropic hormone, or gonadotropins, which act on distinct target organs^2^. Previous studies suggest that most, if not all pituitary cells form structural and functional homotypic cell networks which are highly plastic in response to demands^3^. These cell networks are topologically intermingled and their wiring within the gland is spatially organized both between each other and between each cell network and the rich vasculature which provides the delivery of circulating inputs and hormone clearance into the bloodstream^4^. While communication between distinct intermingled endocrine cell types is of high functional relevance in other endocrine tissues including the pancreas^5^, whether this is also the case in the pituitary is not known. Developmental studies have provided evidence for the interaction of different pituitary cell networks during fetal life^6,7^ and shortly after birth^8^. While it has been shown that the corticotrope network plays an early developmental role in the establishment and organization of the gonadotrope network just before birth^7^, the physiological relevance of inter-network communication in animals of reproductive age has, however, remained unclear.

While corticotropes regulate the secretion of stress hormones from the adrenal cortex, gonadotropes are essential for fertility and provide a functional link between the brain and the gonads by integrating neuroendocrine and steroid hormone signals^9^. Gonadotropes respond to gonadotropin-releasing hormone (GnRH) pulses delivered by neurons in the hypothalamus into the hypophyseal portal blood to secrete gonadotropins, luteinizing hormone (LH), and follicle-stimulating hormone (FSH)^10^. Subsequently, LH and FSH regulate gamete and sex-hormone production by the gonads. Gonadal sex steroids in turn provide direct and indirect feedback to gonadotropes, regulating reproductive cycle control in females.

Recent studies have indicated that in addition to the gonads, other organs including bone, liver, and fat may also be directly regulated by gonadotropins, in particular by FSH^11–13^. Several clinical studies have reported an association of FSH with bone resorption as well as with metabolic disorders including obesity and hepatic steatosis, which occur independently of other hormones^14–16^. Since gonadotropins tightly regulate gonadal function, which itself exerts multiple effects on body homeostasis throughout the whole lifespan of the organism, it has been difficult to experimentally disentangle direct gonadotropin effects on extra-gonadal tissues from indirect effects. Previous studies using either gonadotropin knockout or overexpression mouse strains carrying global genetic modifications reported partially contradicting data in particular regarding FSH actions on extra-gonadal tissues^11,17^.

Here, we combined complementary genetic approaches in mice to demonstrate that acute gonadotrope depletion in adults triggers distinct metabolic disorders including hepatic steatosis and bone loss. We show that while most of these phenotypes are indirect effects due to the hypogonadism caused by gonadotrope ablation, FSH plays a protective role in preventing hepatic steatosis that is independent of the ovary. We uncover paracrine FSH action on corticotropes as a mechanism mediating communication of two distinct cell populations in the pituitary that is essential to restrain the development of hepatic steatosis.

## Results

### Adult female mice develop metabolic disorders upon acute gonadotrope ablation

**Fig. 1:**
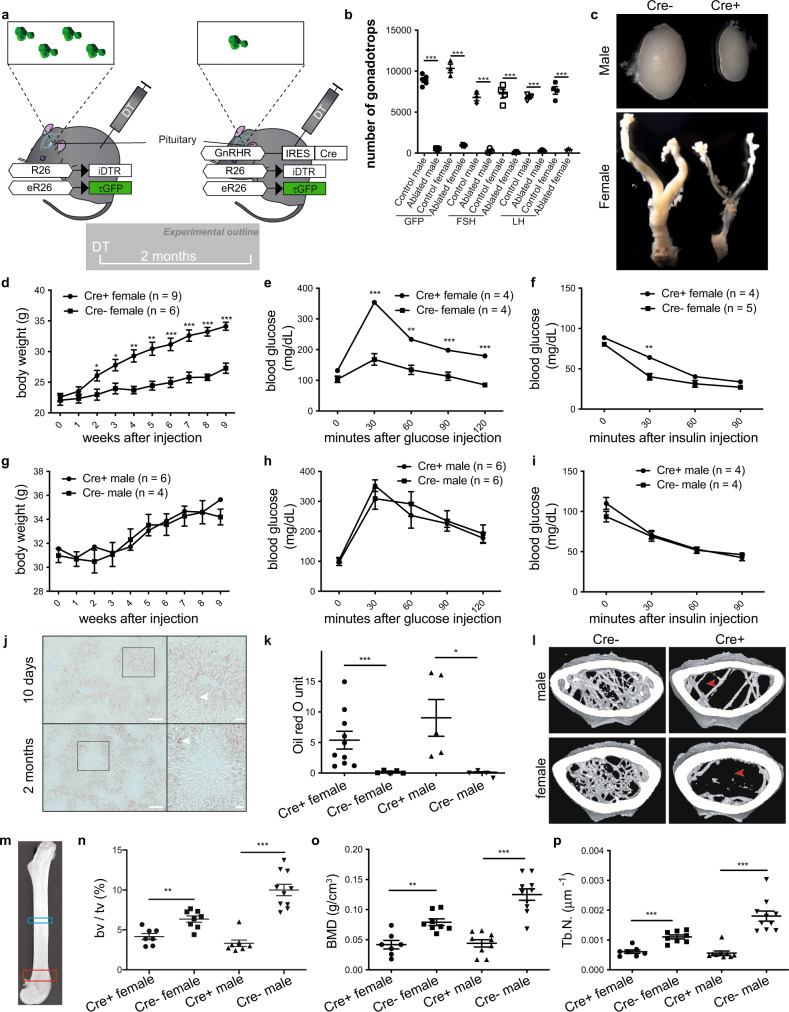
Acute gonadotrope ablation induces metabolic disorders in female mice. **a** Illustration depicting the strategy to acutely ablate gonadotropes via the injection of diphtheria toxin (DT) in mice selectively expressing the diphtheria toxin receptor in gonadotropes. **b** Number of GFP-positive cells within the pituitaries of DT- or saline-injected GRIC/R26-iDTR/eR26-τGFP mice and a number of follicle-stimulating hormone (FSH) or luteinizing hormone (LH) positive cells within the pituitaries of DT-injected Cre+ and Cre− (control) GRIC/R26-iDTR/eR26-τGFP mice. **c** Representative images of testes and female reproductive tracts from Cre+ and Cre− (control) GRIC/R26-iDTR/eR26-τGFP mice 2 months after DT injection. Body weight (**d**, **g**), glucose tolerance (**e**, **h**), insulin tolerance (**f**, **i**), and liver oil red O staining quantification (**k**) from gonadotrope-ablated (Cre+) and control (Cre−) female and male mice. **j** Representative images of liver oil red O staining from Cre+ GRIC/R26-iDTR/eR26-τGFP female mice 10 days or 2 months after DT injection (scale bars = 200 μm. Insets highlight the distribution of lipid droplets (filled arrowheads)). **l** Representative micro-computed tomography (μCT) images from gonadotrope-ablated (Cre+) and control (Cre−) female and male mice (filled arrowheads indicate lack of trabecular bone). **m** Anatomic sites for μCT bone measurements are indicated as blue (cortical bone) and red (trabecular bone) rectangles. Fractional bone volume (**n**), bone mineral density (**o**), and trabecular number (**p**) of trabecular bone from gonadotrope-ablated (Cre+) and control (Cre−) female and male mice. Error bars represent standard error mean. * = *P* < 0.05, ** = *P* < 0.01 and *** = *P* < 0.001. For statistical details, including individual *p*-values, see Supplementary Data 1. Source data are provided as a Source Data file.

To systemically dissect extra-gonadal gonadotropin actions and uncover potential communication between distinct endocrine cell networks within the pituitary, we acutely ablated gonadotropes in adult mice. To do this, we expressed the diphtheria toxin receptor (iDTR) specifically in these cells by generating a mouse strain bearing a gonadotrope Cre recombinase (GRIC)^18^ and Cre-dependent inducible diphtheria toxin receptor (iDTR)^19^ as well as GFP reporter (eR26-τGFP)^20^ alleles (GRIC/R26-iDTR/eR26-τGFP mice; Fig. 1a). Importantly, the iDTR requires the presence of Cre to be expressed but remains inactive in the absence of diphtheria toxin (DT), permitting normal sexual maturation and therefore bypassing developmental effects on the gonads observed in global gonadotropin knockout mice^21,22^. Two months post-DT injection, sexually mature Cre allele-positive (Cre+) adult males and females exhibited a near total ablation of gonadotropes (Fig. 1b) and profound hypogonadism (Fig. 1c), confirming the efficacy of this experimental approach. The numbers of the other hormone-secreting cell types in the anterior pituitary were not affected by gonadotrope ablation (Supplementary Figs. 1 and 2), demonstrating the specificity of our experimental approach. Acute gonadotrope ablation in adults also triggered a dramatic increase in body weight (Fig. 1d), impaired glucose tolerance (Fig. 1e), and decreased insulin sensitivity (Fig. 1f) exclusively in females (Fig. 1d–i), demonstrating sexually dimorphic effects on metabolism upon the severing of functional connectivity between the brain and the gonads. Strikingly, we also observed hepatic steatosis as early as 10 days after gonadotrope ablation in both sexes, which became progressively more severe over time (Fig. 1j, k). Plasma triglycerides were significantly increased in ablated compared with control females, while males showed a possible trend toward an increase but this was not significant (Supplementary Fig. 3). Plasma cholesterol levels were unchanged across all conditions. Next, we analyzed bone phenotypes in gonadotrope-ablated mice, as the bone was previously suggested to be regulated by gonadotropins^11^. We found that bone volume, mineral density, and trabecular number were all significantly reduced in femurs from gonadotrope-ablated mice irrespective of sex (Fig. 1l–p). In addition, cortical bone thickness and bone strength were decreased in female femurs after gonadotrope ablation (Supplementary Figs. 4 and 5). Taken together, these data demonstrate that acute gonadotrope loss in adults recapitulates major constituents of metabolic syndrome in female and, to a lesser extent, male mice.

### Hepatic steatosis in gonadotrope-ablated females is independent of the gonads

**Fig. 2:**
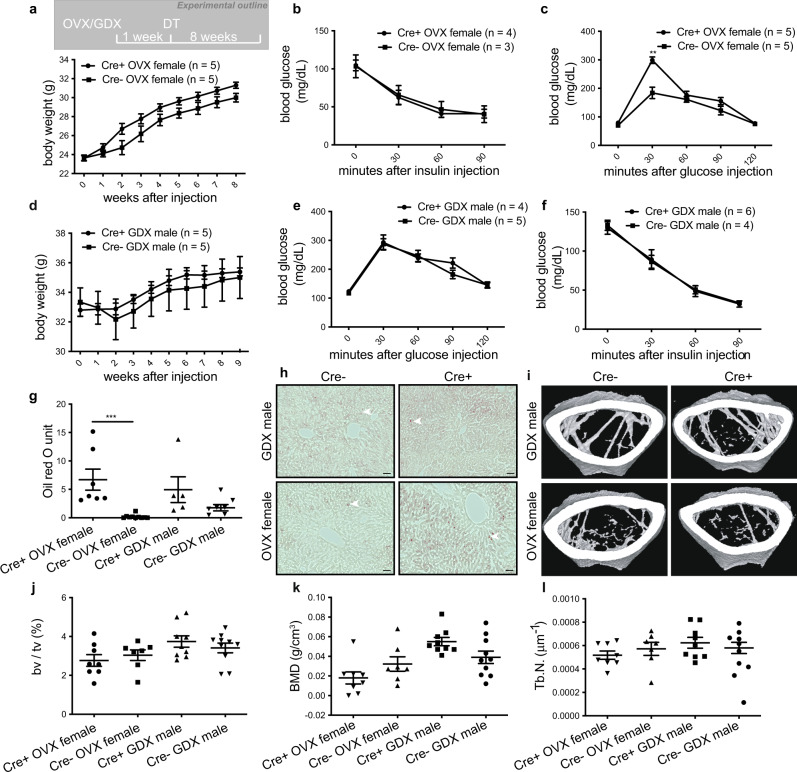
Gonadal loss in females mimics metabolic syndrome but does not trigger hepatic steatosis. **a**, **d** Body weight, glucose tolerance (**b**, **e**), insulin tolerance (**c**, **f**), and liver oil red O staining quantification (**g**) and representative images (**h** scale bars = 50 μm, filled arrowheads indicate lipid droplets.) in gonadotrope-ablated (Cre+) and control (Cre−) ovariectomized (OVX) female and gonadectomized (GDX) male mice. Representative μCT images (**i**), fractional bone volume (**j**), bone mineral density (**k**), and trabecular number (**l**) of trabecular bone from gonadotrope-ablated (Cre+) and control (Cre−) ovariectomized (OVX) female and gonadectomized (GDX) male mice. Error bars represent the standard error of the mean. ** = *P* < 0.01 and *** = *P* < 0.001. For statistical details, including individual *p*-values, see Supplementary Data 1. Source data are provided as a Source Data file.

To dissect the underlying mechanisms, we first asked whether any of the observed phenotypes were mediated by disrupted gonadal function. To address this question, we performed gonadectomy one week prior to diphtheria toxin injection in Cre+ and Cre− mice (Fig. 2a). Ovariectomy followed by DT injection led to a massive reduction of both LH and FSH plasma levels in Cre+ females (Supplementary Fig. 6) and triggered weight gain (Fig. 2a) and decreased insulin sensitivity (Fig. 2b) to a similar extent in Cre+ and Cre− females. Conversely, glucose tolerance was more significantly impaired in ovariectomized Cre+ than Cre- mice (Fig. 2c). Plasma triglycerides and cholesterol levels showed no significant differences between any group (Supplementary Fig. 7). Most notably, in the absence of gonadotrope ablation (Cre−), hepatic steatosis was virtually absent post-ovariectomy. These data demonstrate that the maintenance of systemic glucose homeostasis and hepatic lipid metabolism following the loss of ovarian function depends on intact gonadotropes. In males, no significant differences were found in body weight, insulin sensitivity, or glucose tolerance between castrated Cre+ and Cre− mice after DT injection (Fig. 2d–f); however, both groups developed hepatic steatosis (Fig. 2g, h), indicating that, in contrast to females, fatty liver in males is triggered by the loss of testicular function and revealing an additional sexually dimorphic effect of gonadotrope ablation on metabolism. Gonadectomy alone was sufficient to trigger a bone loss in both sexes, regardless of gonadotrope status (Fig. 2i–l and Supplementary Fig. 8), demonstrating that bone loss is mediated by gonadal dysfunction.

### Sex-steroid replacement rescues the majority of metabolic disorders but not hepatic steatosis in females

**Fig. 3:**
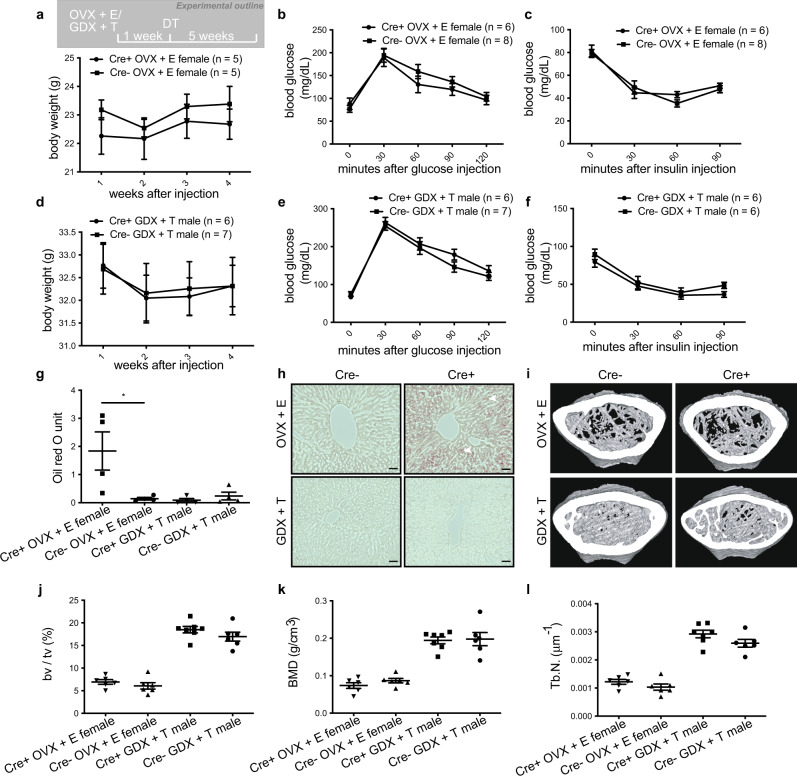
Sex-steroid replacement abolishes metabolic disorders but does not improve hepatic steatosis in gonadotrope-ablated female mice. **a**, **d** Body weight (**a**, **d**), glucose tolerance (**b**, **e**), insulin tolerance (**c**, **f**), and liver oil red O staining quantification (**g**) and representative images (**h**; scale bars = 50 μm, filled arrowheads indicate lipid droplets.) in gonadotrope-ablated (Cre+) and control (Cre−) ovariectomized (OVX) female and gonadectomized (GDX) male mice with estradiol (E) or testosterone (T) replacement, respectively. Representative μCT images (**i**), fractional bone volume (**j**), bone mineral density (**k**), and trabecular number (**l**) of trabecular bone from gonadotrope-ablated (Cre+) and control (Cre−) ovariectomized (OVX) female and gonadectomized (GDX) male mice with estradiol (E) or testosterone (T) replacement, respectively. Error bars represent standard error mean. * = *P* < 0.05. For statistical details, including individual *p*-values, see Supplementary Data 1. Source data are provided as a Source Data file.

To determine whether the phenotypes observed in gonadectomized mice were due to the loss of sex steroids or perhaps other substances released by the gonads, we performed gonadectomy together with hormone replacement followed by gonadotrope ablation (Fig. 3a). Treatment with either estradiol (females) or testosterone (males) abolished differences in glucose tolerance, insulin sensitivity, weight gain, and bone density in gonadectomized mice regardless of sex or whether gonadotropes were ablated (Fig. 3a–f, Fig. 3i–l and Supplementary Fig. 9). Consistent with the bone phenotype we observed, FSH receptor (*Fshr*) and LH receptor (*Lhcgr*) expression were low to undetectable in a murine monocyte cell line differentiated into osteoclasts or in primary murine osteoclasts (Supplementary Fig. 10a–d), suggesting that gonadotropins do not seem to act on bone via osteoclasts. Moreover, FSH did not significantly affect the RANKL-stimulated expression of osteoclast-specific genes (Supplementary Fig. 10e–l) in these cells. Sex steroid replacement also prevented hepatic steatosis in males but had no effect in females (Fig. 3g, h). These data demonstrate that the majority of cardinal features of metabolic syndrome can be precipitated by the absence of gonadal sex steroids; however, uniquely in females, liver steatosis appears to depend on the loss of gonadotropes.

### Gonadotrope activation or FSH administration is sufficient to improve metabolic disorders including hepatic steatosis in female mice

**Fig. 4:**
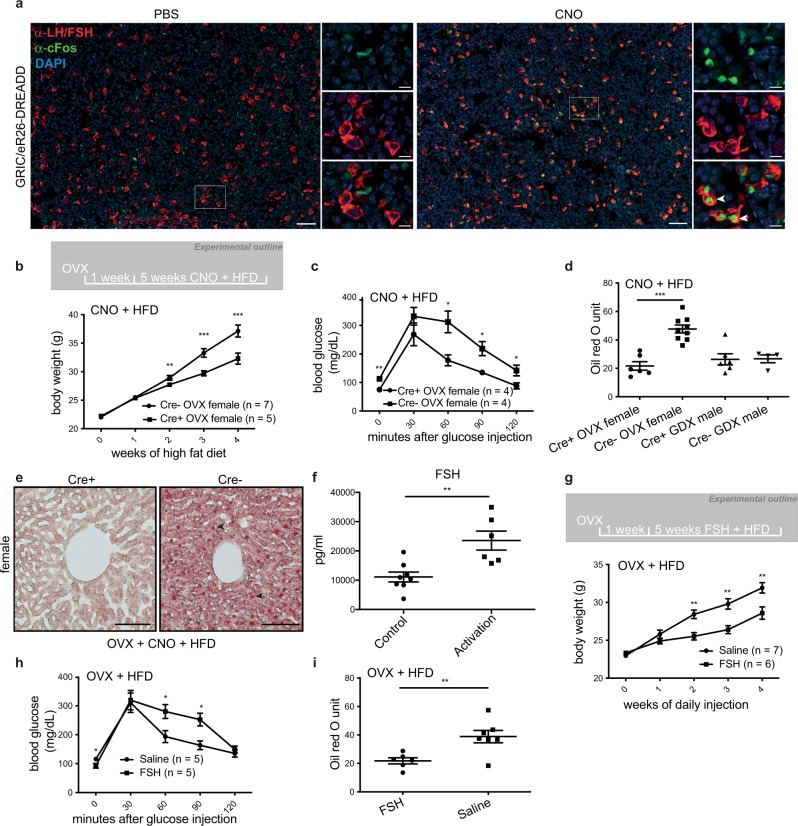
Gonadotrope activation or FSH administration is sufficient to improve metabolic disorders including hepatic steatosis in female mice. **a** Representative images of pituitaries from PBS (left) and clozapine-N-oxide (right) i.p. injected GRIC/eR26-DREADD mice. The expression of cFos (shown in green) was used as a marker for cellular activation. Gonadotropes are shown in red, corresponding to the expression of LH and FSH. DAPI is shown in blue (scale bars = 50 μm in full-size images, 20 μm in insets, and filled arrowheads indicate activated gonadotropes.). **b** Body weight, **c** glucose tolerance, **d** liver oil red O staining quantification, and **e** representative images (scale bars = 100 μm, filled arrowheads indicate lipid droplets) in clozapine-N-oxide (CNO) treated Cre+ (chemogenetically-activated) and Cre− (control) gonadectomized mice on a high-fat diet (HFD). **f** Plasma FSH levels from CNO-injected Cre+ (chemogenetically activated) and Cre− (control) OVX mice on HFD. **g** Body weight, **h** glucose tolerance, and **i** liver oil red O staining quantification in FSH-treated and saline-treated OVX mice on HFD. Error bars represent standard error mean. * = *P* < 0.05, ** = *P* < 0.01 and *** = *P* < 0.001. For statistical details, including individual *P*-values, see Supplementary Data 1. Source data are provided as a Source Data file.

To corroborate these results, we employed a reverse complementary experimental approach and asked whether chronic chemogenetic activation of gonadotropes would relieve the symptoms of metabolic syndrome. To do this, we generated a mouse strain expressing a Gq-coupled DREADD specifically in gonadotropes (GRIC/eR26-DREADD/eR26-τGFP mice; Supplementary Fig. 11a). In this model, clozapine-N-oxide (CNO) triggers robust activation of Gq signaling in gonadotropes, as demonstrated by c-FOS expression (Fig. 4a). We next gonadectomized mice, followed one week later by concomitant administration of CNO in the drinking water and a high-fat diet (HFD; 22% carbohydrates, 24% protein, 54% fat); a well-established model of metabolic syndrome^23^) (Fig. 4b). Remarkably, weight gain was significantly attenuated in female mice with activated gonadotropes relative to controls (Fig. 4b). Likewise, we found that glucose tolerance (Fig. 4c) and hepatic steatosis (Fig. 4d, e) were significantly improved by chronic gonadotrope activation, raising the question as to which gonadotropin might mediate these effects. We, therefore, performed multiplexed hormone analysis on OVX/HFD female mice with chronically administered CNO and found that FSH—but not LH—was significantly increased in response to chemogenetic gonadotrope activation (Fig. 4f and Supplementary Fig. 11b). To determine whether FSH indeed mediates these effects, we performed daily injections of FSH in ovariectomized wild-type mice on HFD. These injections were sufficient to attenuate weight gain (Fig. 4g), improve glucose tolerance, and improve hepatic steatosis (Fig. 4h, i), demonstrating the potential for FSH treatment to improve the symptoms of metabolic syndrome. Plasma triglyceride levels were significantly reduced in the FSH-treated group when compared to controls while cholesterol levels in these animals seemed somewhat lower, however, this was not significant (Supplementary Fig. 12). To gain mechanistic insight into how FSH treatment impinges on the development of liver steatosis, we performed transcriptome analyzes on livers from FSH-treated OVX/HFD females and compared them to control samples. We found differentially expressed genes to be enriched in pathways including cholesterol biosynthesis, FGFR2 ligand binding and activation, and GPCR ligand binding (Supplementary Fig. 13). To gain a detailed insight into transcriptomic changes on lipogenesis, fatty acid oxidation, and lipolysis, we specifically investigated the differentially expressed genes which are involved in lipid metabolism (GO0006629) (Supplementary Fig. 14a). We found that key genes in the control of lipid biosynthesis such as Elovl3, Egr1, Slc45a3^24–26^ were significantly downregulated in the FSH treated group when compared with controls, meanwhile, genes which control lipid catabolism such as Cyp7a1^27^ were significantly upregulated in the FSH treated group when compared with controls, consistent with the fatty livers seen in the FSH treatment group. Interestingly, we also found that genes previously reported to be upregulated by glucocorticoids (dexamethasone) in the liver^28^ were downregulated upon FSH treatment (Supplementary Fig. 14b). These results provide molecular insight into the mechanism underlying liver steatosis in these animals and raise the possibility that FSH may regulate liver steatosis by affecting glucocorticoid signaling in the liver.

### Disrupting FSHR signaling in the pituitary increases corticosterone levels and induces hepatic steatosis

**Fig. 5:**
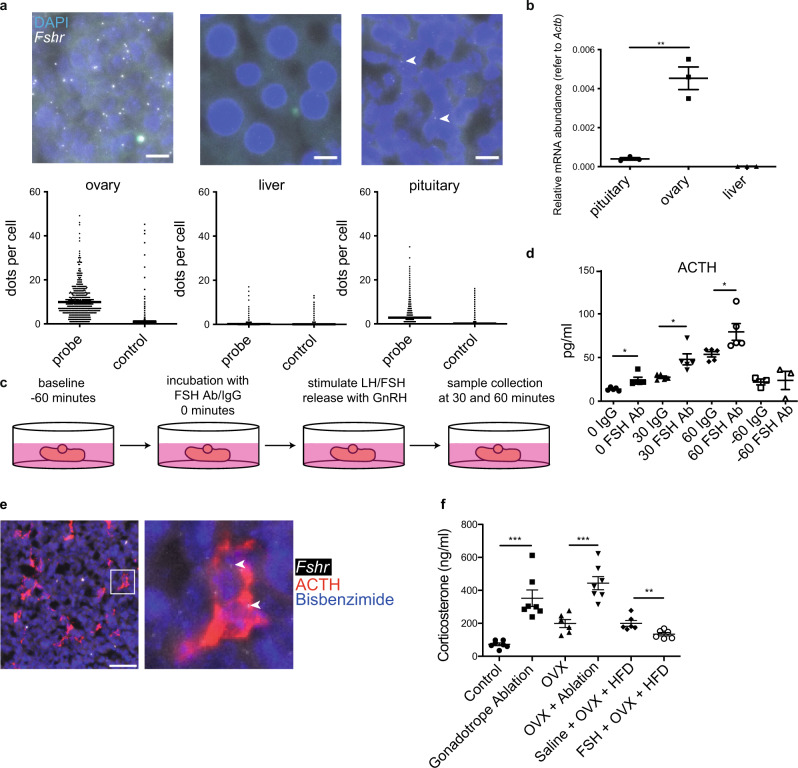
Paracrine FSH action in pituitary corticotropes in female mice. **a** Expression levels of *Fshr* in the pituitary, ovary and liver from adult female mice were measured using RNA scope (scale bars = 10 μm, filled arrowheads indicate positive signals) and **b** RT-qPCR. **c** Schematic representation of in vitro pituitary assay. **d** ACTH levels quantified from in vitro GnRH-stimulated pituitaries incubated with an anti-FSH antibody or IgG (control). **e** Colocalization of *Fshr* mRNA (RNAscope, filled arrowhead) and ACTH (IF) (scale bar = 100 μm). **f** Plasma corticosterone levels from control and experimental female mice. Error bars represent the standard error of the mean. * = *P* < 0.05, ** = *P* < 0.01 and *** = *P* < 0.001. For statistical details, including individual *P*-values, see Supplementary Data 1. Source data are provided as a Source Data file.

To uncover the site of action for FSH in mediating these effects, we performed RT-PCR for the *Fshr* on major tissues prepared from female mice. We detected *Fshr* in only two tissues, the ovary and the pituitary (Supplementary Fig. 15), which we further verified via RNA-scope (Fig. 5a) and RT-qPCR (Fig. 5b), but not in the liver. In addition, a re-analysis of bulk RNA-seq data confirmed that *FSHR* is also not expressed in the human liver (Supplementary Fig. 16)^29^. To understand the role of FSH signaling within the pituitary, we established an in vitro whole pituitary assay in which we blocked the action of FSH by incubating pituitaries taken from diestrus (post-ovulation) females with a monoclonal antibody targeting FSH prior to stimulation with GnRH to release the gonadotropins (Fig. 5c). Among all six of the hormones produced by the anterior pituitary, we found that adrenocorticotropic hormone (ACTH) exclusively was significantly elevated as a result of FSH sequestration by the anti-FSH antibody (Fig. 5d and Supplementary Fig. 17). Vice versa, we also found that ACTH was significantly reduced after chronic gonadotrope activation in vivo (Supplementary Fig. 11b). These findings were consistent with our RNA-scope experiments in which we observed *Fshr* expression in corticotropes (Fig. 5e). Furthermore, re-analysis of pituitary single-cell RNA-seq data^30^ confirmed that *Fshr* is also expressed in human corticotrope cells (Supplementary Fig. 18).

Corticotropes release ACTH to control corticosterone release by the adrenal gland and corticosterone was previously implicated in the regulation of steatosis^28^. We, therefore, hypothesized that FSH reduces corticosterone release via paracrine signaling in the pituitary leading to decreased ACTH release and thus reduced steatosis. Consistent with this, we found significantly elevated ACTH levels in gonadotrope-ablated intact females (Supplementary Fig. 19). ACTH levels after OVX were more variable and also increased, some trend towards further elevated ACTH levels after gonadotrope ablation was, however, not statistically significant. Importantly, gonadotrope ablation either in intact or ovariectomized females resulted in significantly elevated plasma corticosterone concentrations, whereas FSH injections were sufficient to significantly reduce plasma corticosterone levels in ovariectomized mice on HFD (Fig. 5f).

**Fig. 6:**
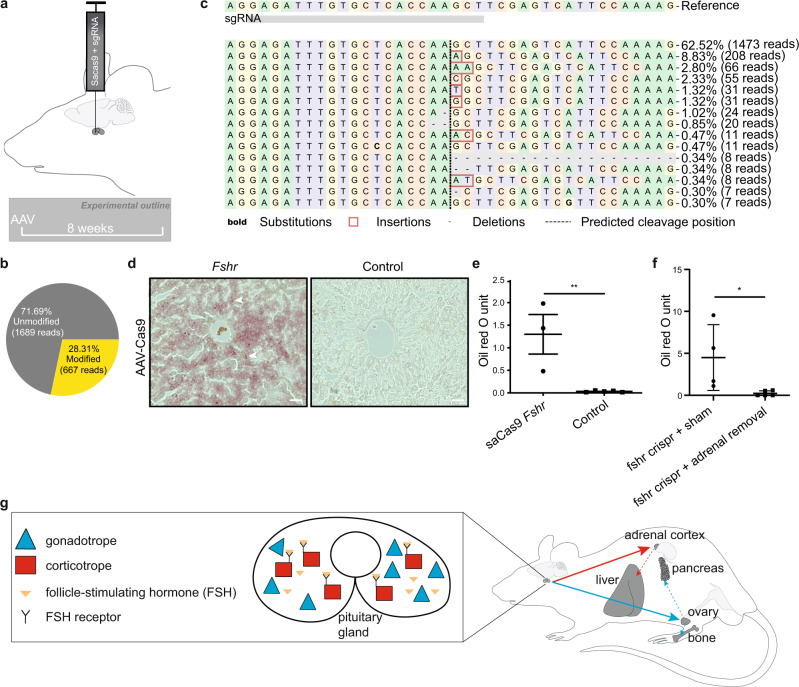
FSH receptor knock-out in the pituitary induces hepatic steatosis in females. **a** Illustration depicting the strategy to specifically disrupt FSH receptor expression in the pituitary via stereotaxic delivery of an adeno-associated virus (AAV) encoding Cas9 and a guide RNA targeting *Fshr* into the pituitary. **b** Percentage of unmodified and modified alleles and **c** distribution of identified alleles around the predicted cleavage site determined by CRISPResso2 of the pituitary DNA from an AAV5-saCas9-*Fshr* injected mouse. **d** Representative images (scale bars = 50 μm) and **e** quantification of liver oil red O staining from AAV-Cas9 (with (*n* = 3 mice) or without (*n* = 5 mice) guide RNA against follicle-stimulating hormone receptor; *Fshr)* pituitary-injected mice. **f** Liver oil red O staining quantification in AAV-Cas9 (with guide RNA against follicle-stimulating hormone receptor; *Fshr*) pituitary-injected adrenalectomized or sham-operated female mice. **g** Model of gonadotrope actions on the gonads and the pituitary (filled blue arrow illustrates gonadotrope actions on the gonads; dashed blue arrows indicate indirect gonadotrope effects on pancreas and bone via sex steroids released by the gonads; filled red arrow indicates corticotrope action on the adrenal cortex (via paracrine FSH intra-pituitary action); dashed red arrow indicates the effect of elevated corticosterone levels on the liver). Error bars represent the standard error of the mean. ** = *P* < 0.01. For statistical details, including individual *P*-values, see Supplementary Data 1. Source data are provided as a Source Data file.

To functionally analyze paracrine FSH action, we used a CRISPR–Cas9 system to specifically disrupt FSH receptor expression in the pituitary. We stereotaxically injected an adeno-associated virus (AAV) to deliver Cas9 in combination with a guide RNA targeting *Fshr* into the pituitary (Fig. 6a). Strikingly, even partial knockout of *Fshr* (Fig. 6b, c) within the pituitary was sufficient to trigger hepatic steatosis in AAV-injected female mice, when compared to controls (Fig. 6d, e). In contrast, *Fshr* knockout within the female pituitary after adrenalectomy did not result in a fatty liver phenotype (Fig. 6f). Consistent with this, we found corticosterone levels to be elevated in the adrenal-intact pituitary-specific *Fshr* knockout females (Supplementary Fig. 20). Taken together, these data demonstrate that paracrine FSH action on corticotropes in the pituitary regulates hepatic lipid metabolism by reducing ACTH and subsequently corticosterone secretion.

## Discussion

To analyze extragonadal gonadotropin action(s), we combined complementary genetic strategies to manipulate gonadotrope cells in mice. These experiments yielded several important results. First, adult female but not male mice develop metabolic disorders upon acute gonadotrope ablation. Second, hepatic steatosis in gonadotrope-ablated females is independent of the gonads. Third, sex-steroid replacement rescues the majority of metabolic disorders but not hepatic steatosis in females. Fourth, gonadotrope activation or FSH administration is sufficient to improve metabolic disorders including hepatic steatosis in female mice. Finally, disrupting FSHR signaling in the pituitary increases corticosterone levels and induces hepatic steatosis. Taken together, our data uncover paracrine FSH action within the pituitary gland as a mechanism to restrain the development of hepatic steatosis.

While we show here that gonadotropes act via paracrine FSH action on corticotropes in adults, previous experiments had suggested that the establishment of the corticotrope network controls the anatomical organization of the gonadotrope network during fetal development^7^, raising the possibility that corticotropes communicate with gonadotropes in utero. Functional communication between these two endocrine cell populations may also provide an explanation for why gonadotropes and corticotropes are intermingled and invariantly positioned in close proximity to each other in the gland^7^. FSH plays a protective role in the liver restraining the development of hepatic steatosis through a previously unanticipated paracrine mechanism in the pituitary. These data provide clear evidence in the pituitary in vivo for an emerging endocrine paradigm; the structural and functional organization of one endocrine cell type can impinge on the functioning of other endocrine cells within the same gland. Other important examples include pancreatic islets in which alpha glucagon cells act on beta cells^5^.

Functional communication between gonadotropes and corticotropes might represent the first of several paracrine systems in the pituitary. Consistent with this, re-analysis of human pituitary single-cell RNA-seq data^30^ confirmed the expression of heterotypic hormone receptors in the distinct endocrine pituitary cell types (Supplementary Fig. 21). Our data now set the stage to look at communication between the cell networks producing growth hormone, prolactin or thyroid-stimulating hormone in the anterior pituitary gland. Future experiments will also need to address the question of whether FSH also acts on endocrine pituitary cell networks other than corticotropes. Progress analyzing inter-network communication should also be facilitated by the technical advances presented here including stereotactic injections of Cre-dependent AAVs carrying specific guide RNAs into the pituitary to achieve gene conditional knockouts in this tissue representing a major temporal advantage over classical gene targeting techniques^31^. Furthermore, an adaptation of this technique to include several guide RNAs should allow the knockout of multiple genes simultaneously, providing an additional temporal and spatial advantage and also allowing a reduction of the number of animals needed for multiple gene knockouts.

Future studies will address the mechanism(s) underlying the sexually dimorphic effects on metabolism (increase in body weight, impaired glucose tolerance, and decreased insulin sensitivity in females, but not in males) upon acute gonadotrope ablation in adults. Why does FSH not regulate hepatic steatosis in male mice? To address this question, we analyzed *Fshr* expression in males. Strikingly, and in contrast to females, we did not detect *Fshr* expression in the pituitary in males (Supplementary Fig. 22), suggesting that FSH does not exert paracrine actions in males and providing a molecular mechanism explaining why FSH does not regulate hepatic steatosis in male mice.

Our data do not support the previous model of FSH action in bone. Instead, by using complementary approaches in mice, we demonstrate unequivocally that gonadotropins influence bone metabolism indirectly via sex steroid production and not by direct action on bone. Loss of sex hormones was previously described to contribute to bone loss; however, direct FSH effects on bone were debated^32,33^. While osteoporosis after gonadectomy had been attributed to decreased sex hormone levels^34^, several studies challenged this view by reporting that FSH directly regulated bone metabolism. Global FSHβ or FSHR knock-out in female mice resulted in hypogonadism without bone loss, consistent with a protective role for FSH on bone^11^. Combined with in vitro studies, the authors speculated that this effect of FSH on bone metabolism is direct and independent of estrogen. One caveat we need to consider is that FSHβ or FSHR were removed in the germline in these animals. Since FSH signaling is one key regulator of sexual maturation, a global knock-out of FSHβ or FSHR could have developmental effects including compensation in these models. Therefore, the reported bone phenotype could for example result from elevated LH and/or testosterone levels as reported^35,36^.

Reproductive axis dysfunction had previously been implicated in the development of metabolic disorders. For example, in women with polycystic ovary syndrome (PCOS), aberrantly elevated LH secretion results in anovulation and hyperandrogenism, as a result of elevated testosterone production by ovarian theca cells^37^. Nearly half of all women with PCOS are affected by metabolic disorders^38^ including hyperinsulinemia and insulin resistance and nonalcoholic fatty liver disease (NAFLD), however, whether PCOS is the result of metabolic disorders or vice versa PCOS triggers metabolic disorders is still not well understood. The acute ablation of gonadotropes in adults, which shuts down the HPG axis, clearly induced metabolic disorders in this experimental setup. This may provide insights to understand the association between reproductive disorders, including PCOS, and metabolic disorders.

Furthermore, men with congenital testosterone deficiency as a result of Klinefelter syndrome (47, XXY) are four times more likely to develop metabolic syndrome^39^. Suppressing the reproductive axis in men using GnRH analogs, as for the treatment of prostate cancer, triggers weight gain, bone loss, and insulin resistance^40,41^. Our findings that FSH injection reduces weight gain and improved glucose tolerance in the absence of either ovarian or supplemented estradiol highlight the potential clinical benefit of FSH receptor agonists for the treatment of metabolic syndrome and also open up the possibility of drug repurposing for FSH.

## Methods

### Generation of the Rosa26-NLSiRFP720-2A-Gq (eR26-DREADD) knock-in mice

DREADD mice were generated by homologous recombination in mouse embryonic stem (ES) cells using a targeting construct designed to insert a CAGS promoter (CMV enhancer plus chicken ß-actin promoter)-driven NLSiRFP720-2A-Gq receptor (DREADD receptor) within the first intron of the *Rosa26* gene locus. This encodes both an infrared fluorescent protein, which is directed to the cell nucleus, and a Gq-coupled receptor, which can be specifically activated by CNO administration. To ensure that this expression is Cre-dependent, floxed strong transcriptional stop signals (three SV40 polyA signals) are present in such a way that the CAGS promoter can only drive expression following Cre-dependent removal of the stop signals. Correct insertion of the NLSiRFP720-2A-Gq receptor construct was verified using Southern blot analysis as follows. DNA was extracted from tail tip biopsies using lysis buffer containing 0.1 mg/mL proteinase K (1 mg/mL was used for extraction from ES cells). Following extraction, genomic DNA was digested overnight with *EcoR*I and run on a 0.7% agarose gel, then transferred to a nylon membrane by capillary transfer and screened by hybridization of a 491 bp ^32^P-labeled probe complementary to sequences located 5′ to the 5′ homology arm of the targeting construct. Probe hybridization produces a 15.6-kb band from the wild-type allele, whereas the correctly targeted allele generates a 5.8-kb band. Correctly targeted ES cells were injected into C57BL/6J blastocysts to generate male chimeras that were backcrossed to C57BL/6J females to produce heterozygous *Rosa26*-NLSiRFP720-2A-Gq mice. Mice were then further crossed to produce a homozygous colony.

Homozygous Rosa26-NLSiRFP720-2A-Gq mice were crossed with appropriate Cre-expressing lines to generate offspring in which specific cell populations can be activated by CNO. Mice were maintained on a mixed genetic background of 129S × C57BL/6J. The genotypes of the Rosa26-NLSiRFP720-2A-Gq mice were confirmed by PCR using the primer sequences: 5-GGAAGCACTTGCTCTCCCAAAG-3′ (common forward primer); 5′-GGGCGTACTTGGCATATGATACAC-3′ (DREADD allele reverse primer) and 5′-CTTTAAGCCTGCCCAGAAGACTC-3′ (wildtype allele reverse primer). Wild-type offspring were confirmed by the presence of a single band of 256 bp. For the Rosa26-NLSiRFP720-2A-Gq allele, heterozygous offspring gave two products of 256 and 495 bp, whereas homozygous offspring were identified by the presence of one band at 495 bp.

### Mice

All mice were kept under SPF housing with food (V1534-300, Ssniff) and water ad libitum. Animal care and experimental procedures were conducted under the approval of the animal welfare committees of Saarland University and Xi’an Jiatong University. Mice were monitored on a daily basis by trained personnel. To label and ablate or pharmacologically activate *Gnrhr*-expressing cells, we used the GnRHR-IRES-Cre (GRIC) knock-in mouse strain^18^ crossed either with eROSA26-τGFP (eR26-τGFP)^20^ and ROSA26-DTR (R26-DTR)^19^ animals, or with eROSA26-τGFP (eR26-τGFP) and eROSA26-DREADD (eR26-DREADD) animals. In the resulting GRIC/eR26-τGFP/R26-DTR or GRIC/eR26-tGFP/eR26-DREADD mice, Cre recombinase is expressed under the control of the *Gnrhr* promoter. Cre-mediated recombination results in the removal of a transcriptional stop cassette from the ROSA26 locus and subsequent constitutive reporter expression in GnRHR cells. All mice were kept on the same mixed 129 × C57BL/6J genetic background.

### Gonadectomy and sex hormone replacement

Adult mice (8–12 week-old female mice or 14-16 week-old male mice) were bilaterally ovariectomized or castrated. For sex hormone replacement, a 2 cm length of silastic tubing (inner diameter: 1.58 mm; outer diameter: 3.18 mm) containing 36 μg 17β-estradiol/mL (E2758, Sigma)^42^ in sesame oil or testosterone powder^43^ (T1500, Sigma) were inserted on the dorsal aspect of the animal’s neck immediately after gonadectomy. Mice were allowed to recover for 1 week before further treatment.

For ablation experiments, 8- to 10-week-old female mice or 14- to 16-week-old male mice were intraperitoneally injected with 20 ng/g bodyweight diphtheria toxin (DT; 322326, EMD Millipore) twice with a 3-day interval between injections. For activation experiments, 2.5 mg of clozapine-N-oxide (CNO; Tocris Bioscience) in 200 ml of drinking water was administered to the mice with a high-fat diet (22% carb, 24% protein, 54% fat, E15742-347, Ssniff). For FSH administration, mice with a high-fat diet received daily 30 IU/kg i.p FSH (GONAL-f, Merck) injections. Mice were weighed and monitored daily before being euthanized as experimental mice. Mice were humanely euthanized if postsurgical complications progressed to a pre-defined humane endpoint at which the mice started to suffer.

### Glucose and insulin tolerance test

An intraperitoneal glucose tolerance test (ipGTT) was performed in mice fasted for 16 h. Briefly, the blood was collected from the tip of the tail and blood glucose was measured before (0 min) and after (30, 60, 90, and 120 min) glucose administration (2 g/kg body weight, i.p.) using a digital glucometer (Accu-Check Performa). Intraperitoneal insulin tolerance test (ipITT) was performed in mice fasted for six hours and the blood glucose was measured before (0 min) and after (30, 60, and 90 min) insulin administration (Pharma Gerke Arzneimittelvertrieb) 0.5 U/kg body weight, i.p.

### Transcardial perfusion and immunostaining

Mice were perfused, and tissues were sectioned as previously described except cardiac puncture was performed to harvest blood for plasma preparation prior to PBS perfusion^44^.

### Immunohistochemistry

Immunostaining was performed as previously described^44^. In brief, pituitaries were sectioned with 14 μm thickness, and every 10th section was stained. Antisera used were as follows: rabbit anti-LH (1:5000, National Institute of Diabetes and Digestive and Kidney Diseases (NIDDK)), guinea pig anti-FSH (1:5000, NIDDK), guinea pig anti-ACTH (1:1000, NIDDK), guinea pig anti-GH (1:1000, NIDDK), guinea pig anti-TSH (1:1000, NIDDK), rabbit anti-prolactin (1:1000, NIDDK), rabbit anti-c-Fos (1:500, #2250, Cell Signaling Technology), chicken anti-GFP (1:1000, #A10262 ThermoFisher), goat anti-chicken Alexa 488 (1:500, Invitrogen, #A11039), goat anti-rabbit Cy3 (1:500, Jackson ImmunoResearch, #711-165-152) and goat anti- guinea pig Cy5 (1:500, Jackson ImmunoResearch, #706-175-148). Images were captured using a ZEISS Axio Scan Z1. The number of positive cells was multiplied by 10 to estimate the cell number in the whole pituitary.

### RNA-seq

Transcriptomic analyses were performed as previously described^45^. In brief, total RNA from liver tissue was purified by using the RNeasy Plus Mini kit (QIAGEN) according to the manufacturer’s instructions. RNA samples with an RNA integrity number greater than eight were used to build RNA-seq libraries. One microgram of total RNA for each sample was used to build libraries employing the NEB Next Ultra RNA Library preparation kit (Ipswich). The library was sequenced in the Illumina Hiseq2500 platform with 2× 100-bp paired-end reads. After alignment, transcripts with an absolute value of log2 (fold change) larger than 1 and a *q* value below 0.05 were considered to be differentially expressed. Gene Set Enrichment Analysis (GSEA) was performed to functionally study the transcriptomic changes.

### RNAscope in situ hybridization

*Fshr* mRNA expression was assessed in the pituitary, liver, and ovary from adult female mice using RNAscope 2.5 High-definition Assay-RED (Advanced Cell Diagnostics, Hayward, CA), according to the manufacturer’s instruction as previously described^46^. The *Fshr* probe used is designed to target transcript NM_013523.3 with a target sequence spanning nucleotides 554–1487. Probes targeting the DapB gene from *Bacillus subtilis* were used as a negative control. In brief, endogenous peroxidase activity was blocked with an RNAscope hydrogen peroxide solution. Tissues were permeabilized with RNAscope protease plus. Sections were then hybridized with either the *Fshr* probe or the negative control probe. This was followed by a series of amplification incubation steps. Finally, the hybridization signals were detected by detection reagents. Nuclei were stained with bisbenzimide. Images were captured using a ZEISS Axio Scan Z1. Positive hybridization was determined using Advanced Cell Diagnostic’s RNAscope scoring guidelines. To determine the colocalization of *Fshr* mRNA and ACTH, immunolabelling of ACTH was performed on the same pituitary sections after RNAscope in situ hybridization.

### μCT analysis

Femora were dissected from mice, stored at −80 °C, and then scanned by high-resolution μCT (Skyscan 1176, Bruker MicroCT, Kontisch, Belgium). The scanner was set at a voltage of 50 kV, a current of 200 μA, and an isotropic resolution of 9 μm by using a 0.5 mm aluminum filter. We used image reconstruction software (NRecon (version 1.6.10.6)), orientation software (DataViewer (1.5.1.2)), data analysis software (CTAn (version)1.16.4.1+), and three-dimensional model visualization software (CTVox (version 3.2.0r1294)) in order to analyze the parameters of the distal femoral metaphyseal trabecular bone. Trabecular bone was analyzed over 200 slices, starting with 50 slices distal from the growth plate. The trabecular bone volume fraction (BV/TV) and trabecular number (Tb.N) were analyzed for trabecular bone. The cortical bone thickness was analyzed for cortical bone. Bone mineral density was estimated using calcium hydroxyapaptite (CaHA) phantoms of known densities.

### Cloning and guideRNA selection

Potential guideRNA (gRNA) sequences were identified using Chopchop^47^ for the *Fshr* gene. From the identified gRNAs, an optimal sequence was selected to have the lowest possibility of off-target events whilst having a high predicted efficiency and frameshift likelihood at the targeting site after analysis with Cas-OFFinder^48^ and CRISPOR^49^. The following gRNA was selected for targeting; *Fshr*: 5′-GAGATTTGTGCTCACCAAGCT. This gRNA was generated as a primer dimer and then ligated into pX601-AAV-CMV (a gift from Feng Zhang^50^; Addgene plasmid #61591) using the *Bsa*I sites present within this plasmid. This inserted the gRNA sequence subsequent to a U6 promoter and immediately upstream of the gRNA scaffold sequence required for correct interaction with the Cas9 protein. The saCas9 sequence was also present within this plasmid under the control of the strong CMV enhancer and promoter. Following cloning, all elements were verified by sequencing of the entire region contained between the two ITR sites. For the production of a control virus, the unmodified pX601-AAV-CMV plasmid without gRNA was used.

### AAV vector production

Both the gRNA and control viruses were produced using the triple transfection helper-free method. This involved transfecting HEK293T cells in culture with 3 plasmids in a 1:1:1 ratio, the first containing essential viral genes such as *E2* and *E4* (pAdDeltaF6 was a gift from James M. Wilson; Addgene plasmid # 112867), the second which facilitates the generation of serotype 5 AAV vectors (pAAV2/5 was a gift from Melina Fan; Addgene plasmid # 104964) and the third, which dictates the packaged contents of the virus particles. Transfection was undertaken when the cells reached 60-70% confluency using a 4:1 (v:w) ratio of Polyethylenimine (PEI) to plasmid DNA. Sixty to seventy-two hours after transfection, cells were pelleted and processed to recover the virus. Virus samples were then subjected to purification through an iodixanol gradient before desalting and concentration using a centrifugal filter (MWCO 100). Viral titer was measured by qPCR analysis with primers specific to the ITR region of the packaging plasmid (fwd ITR primer: 5′-GGAACCCCTAGTGATGGAGTT, rev ITR primer: 5′-CGGCCTCAGTGAGCGA).

### Stereotaxic injection

Stereotaxic injections were performed as previously described^44^. Briefly, six injections with 0.5μl per injection of AAV virus (AAV5-saCas9-*Fshr* or AAV5-saCas9) were injected into the pituitary. The coordinates were −2.55 mm, −2.7 mm, and −2.95 mm antero-posterior, ±0.6 mm lateral to the midline, 300 µm above the sella turcica. Mice were humanely euthanized if postsurgical complications progressed to a pre-defined humane endpoint at which the mice started to suffer.

### Adrenalectomy

Bilateral adrenalectomy was performed in wild-type adult female mice. The adrenalectomized animals were supplied with 0.9% saline to maintain salt levels. One week after surgery, the AAV virus (AAV5-saCas9-*Fshr*) was stereotaxically injected into the pituitary to knock down the expression of *Fshr*. Mice were humanely euthanized if postsurgical complications progressed to a pre-defined humane endpoint at which the mice started to suffer.

### Oil red O (ORO) staining

Liver tissue sections at −80 °C were first allowed to reach room temperature over 30 min. Then the slides were incubated with 60% (v/v) isopropanol for five minutes, followed by 10 min incubation with fresh ORO (Oil Red O, O0625, Sigma) working solution (7:5 dilution of ORO stock solution with water; ORO stock solution: 300 mg ORO in 100 ml 100% isopropanol). Then two washing steps for three minutes in 60% isopropanol with 100 rpm shaking were performed. After three washing steps of one minute each in water, slides were mounted with Fluormount G onto Superfrost Plus microscopy slides. For visualization, slides were imaged using a ZEISS Axio Scan Z1 with ZenBlue software (supported by the DFG INST 256/434-1 FUGG) with 20× magnification. We noticed ORO signals were not evenly distributed in sections. Therefore at least three complete sections per mouse liver were stained and the ORO units (ORO area/tissue area * 100) were calculated by Image J as previously described^51^.

### In vitro pituitary assay

Intact pituitaries were removed from adult females at diestrus, then incubated with 1 ml DMEM in a 12-well plate at 37 °C, 5% CO_2_, with constant shaking for 1 h. After resting, pituitaries were incubated with 1 ml of fresh DMEM with either mouse monoclonal FSH antibody (10 μg/ml; MIF2709, Invitrogen) or mouse IgG (10 μg/ml; MAB002, R&D Systems) for 1 h. 50 μl of culture medium was taken as a 0-time point. Then pituitaries were stimulated with 100 nM GnRH (L7134, Sigma-Aldrich). Fifty microlitres of culture medium were removed at each time point.

### Hormone measurements

Pituitary hormone measurements were performed with a Milliplex MAP mouse pituitary magnetic bead panel (RPTMAG-86K; Millipore, Billerica, MA) on a Luminex Magpix (Austin, TX) with Milliplex Analyst software according to the manufacturer’s protocol.

### Corticosterone measurements

Circulating corticosterone was measured via colorimetric ELISAs according to the manufacturer’s protocol (ab10882, Abcam). The assays were done in duplicate with an inter and intra-assay variability of 10.6% and 6.3%, respectively.

### CRISPR efficiency verification

Eight weeks after the AAV injection, pituitaries and livers were removed from the mice. DNA was extracted from pituitaries and then targeted amplification was performed via PCR with the primers against *Fshr* (primers used in PCR are described in Supplementary Table 1). Amplicons were generated using region-specific primers with the Illumina universal adapter sequences. PCR products were purified with Agencourt AmpureBeads and indexed in a second PCR using Illumina TruSeq adapters. After the final AmpureBead Purification amplicons were pooled in an equimolar ratio and sequenced on a MiSeq (Illumina) using the MiSeq Reagent Kit v2 (500-cycles) in paired-end mode, aiming at 10,000 reads per amplicon. The sequence was then analyzed by CRISPResso2^52^.

### Osteoclast differentiation in RAW 264.7 cells and primary murine monocytes

RAW 264.7 cells (ATCC TIB-71) were seeded in 96-well plates at a density of 1000 cells/well in Dulbecco’s Modified Eagle Medium (DMEM, Wisent Inc, 319-005-CL), containing 10% fetal bovine serum (FBS), and 1% Antibiotic-Antimycotic solution (ThermoFisher Scientific, 15240062) at 37 °C/5% CO_2_ in a humidified water-jacketed incubator. After overnight incubation, cells were treated with 0, 35, 70, or 140 IU/L human FSH (hFSH, 17.5 IU/μg, R&D Systems, 5925-FS-010) in the presence of 50 ng/mL receptor activator of nuclear factor κ B ligand (RANKL, Peprotech, 315-11) for seven days. The medium was changed every other day.

Femora and tibiae from *Fshr*^fx/fx^ female mice^53^ were collected for bone marrow cell extraction closely following a standard protocol^54^ adapted for 48-well plates. Briefly, the animals were euthanized and one animal was used per experimental replicate. Femora and tibiae were collected and kept in 1× phosphate-buffered saline (1X PBS, Wisent Inc., Canada) on ice. Small cuts (approximately 1–2 mm) were made at both the proximal and distal ends of the bones before placing each in a 200 μL tip with 2 cm removed from both ends within 1.5 mL tubes. Tubes were centrifuged at 10,000×*g* for 15 s at room temperature. Bone marrow was pulled to the bottom of the tubes before being transferred to a 50 mL tube with 3 mL filtered RBC lysis buffer (Geneaid, Taiwan) and incubated at room temperature for 5 min. The reaction was stopped by adding 27 mL 1× PBS and tubes were centrifuged at 500×*g* for 5 min at room temperature. Supernatants were aspirated and cells were resuspended in 10 mL Alpha Modified Eagle’s Medium (AMEM; Wisent Inc., Canada) containing 10% FBS, 1% Antibiotic–Antimycotic solution, and 25 ng/mL M-CSF (Peprotech, USA) prior to culturing in 10 cm dishes. After overnight incubation, media with non-adherent cells (enriched in the myeloid lineage) were collected and seeded in 48-well plates at a density of 2 × 10^5^ cells/well with 25 ng/mL M-CSF in the absence or presence of 50 ng/mL RANKL and 0, 70, or 140 IU/L hFSH for 4 days. Differentiation media were refreshed on day 3.

### Reverse transcription and real-time PCR

To analyze *Fshr* expression in mouse tissues, total RNA was purified from adult female tissues using the RNeasy Plus Micro kit (Qiagen) according to the manufacturer’s instructions. For reverse transcription PCR, genomic DNA removal and cDNA synthesis were performed using a Maxima H Minus First Strand cDNA-Synthesis kit (Thermo Fisher). PCR was performed using a MyTaq Red Mix kit (Bioline). Real-time PCR was performed using a SensiFAST SYBR No-ROX one-step kit (Bioline) with a CFX-96 real-time PCR detection system (Bio-Rad Laboratories) as described previously^45^.

To examine gene expression in cultured cells, total RNA from cells was extracted in TRIzol (Life Technologies, USA; Catalog No. 15596026) following the manufacturer’s instructions. 200 ng of RNA per sample was used for reverse transcription. Quantitative PCR was performed on a Corbett Rotorgene 600 instrument (Corbett Life Science) using BrightGreen 2× qPCR MasterMix (ABM, Mastermix-S). Primers used in reverse transcription and real-time PCR are described in Supplementary Table 1.

### Statistical analysis

GraphPad Prism was used to perform the statistical analysis. Data are presented as mean ± standard error of the mean. For body weight, GTT, and ITT experiments, a two-tailed Student’s *t* test followed by Wilcoxon signed-rank test was performed for individual time points. For two group comparisons, a two-tailed Student’s *t* test was used. Statistical details are described in Supplementary Data 1.

### Reporting summary

Further information on research design is available in the Nature Portfolio Reporting Summary linked to this article.

## Supplementary information

Supplementary Information

Description of Additional Supplementary Files

Supplementary Data 1

Reporting Summary

## Data Availability

Raw sequencing data have been deposited on GEO under accession code GSE216096. The published single-cell RNA-seq data from human pituitary re-used in this study were obtained from GEO through the accession code GSE142653^29^. All other data generated or analyzed during this study are included in this published article (and its supplementary information files). Source data are provided in this paper.

## Source data

Source Data

## Code availability

The custom ImageJ plugin that supports the findings of this study is available in Zenodo with the identifier 10.5281/zenodo.7197582 [https://zenodo.org/record/7197582#.Y712SRWZOUk].
