# Supplementary material for: Intra-pituitary follicle-stimulating hormone signaling regulates hepatic lipid metabolism in mice
Source: Nat Commun. 2023 Feb 25;14:1098. doi: 10.1038/s41467-023-36681-z (PMC9968338; doi:10.1038/s41467-023-36681-z)
Supplement: Supplementary file 1 — Supplementary Information [file 41467_2023_36681_MOESM1_ESM.pdf]

Supplementary Figure 1

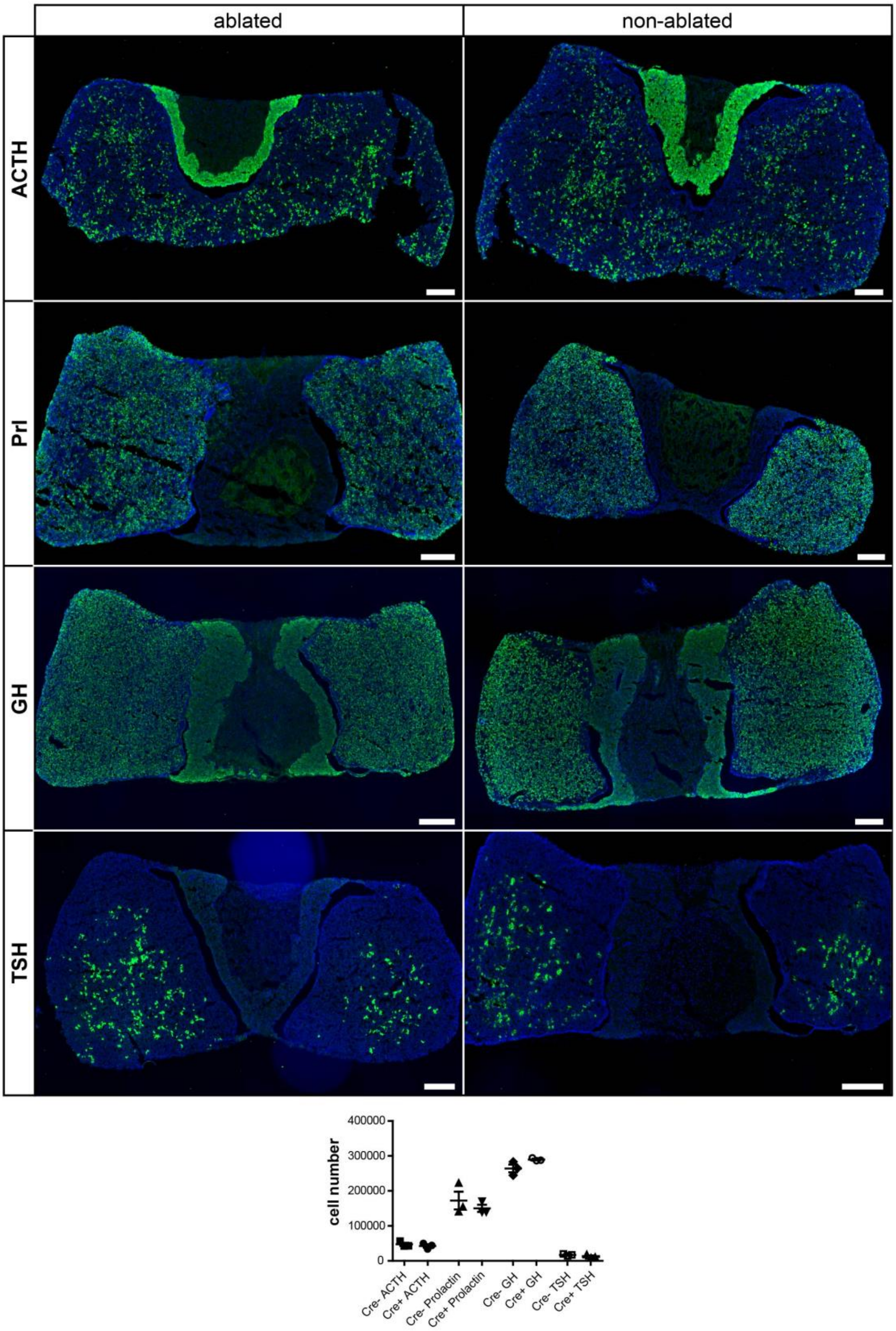

Supplementary Figure 1. Gonadotrope ablation does not affect other hormone-secreting cell types in the male anterior pituitary gland. Pituitary hormones (ACTH, adrenocorticotropic hormone; Prl, prolactin; GH, growth hormone; TSH, thyroid-stimulating hormone) were detected in male gonadotrope-ablated (ablated) and control (non-ablated) pituitaries by immunofluorescence analyses (green). Nuclear stain is shown in blue. Scale bars: 200 µm. Bottom: Cell numbers were not significantly different between ablated (n=3 animals for each hormone) and non-ablated animals (n=3 animals for each hormone). Error bars represent the standard error of the mean. For statistical details, see Supplementary Data 1. Source data are provided as a Source Data file.

Supplementary Figure 2

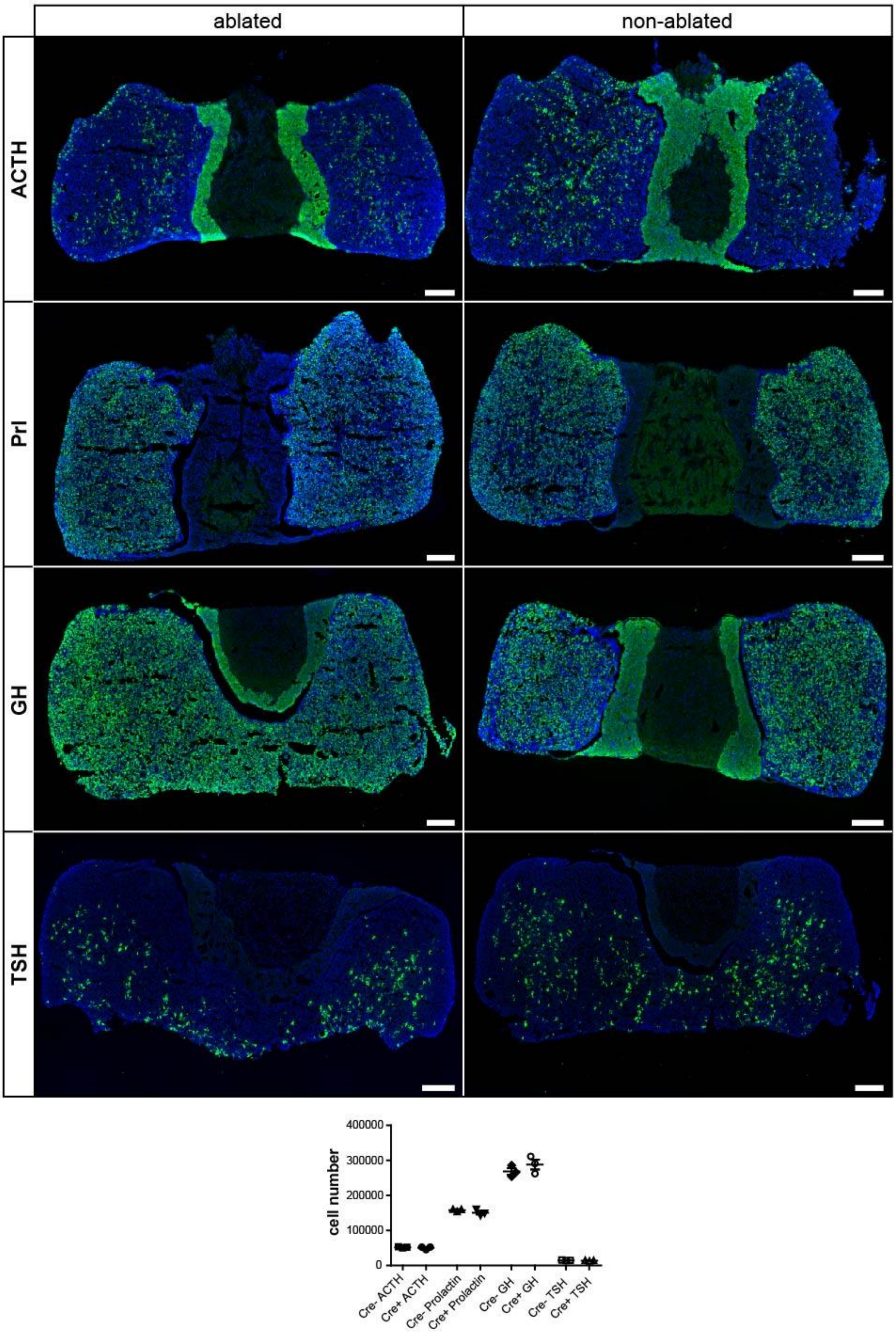

Supplementary Figure 2. Gonadotrope ablation does not affect other hormone-secreting cell types in the female anterior pituitary gland. Pituitary hormones (ACTH, adrenocorticotropic hormone; Prl, prolactin; GH, growth hormone; TSH, thyroid-stimulating hormone) were detected in female gonadotrope-ablated (ablated) and control (non-ablated) pituitaries by immunofluorescence analyses (green). Nuclear stain is shown in blue. Scale bars: 200 µm. Bottom: Cell numbers were not significantly different between ablated (n=3 animals for each hormone) and non-ablated (n=3 animals for each hormone) animals. Error bars represent the standard error of the mean. For statistical details, see Supplementary Data 1. Source data are provided as a Source Data file.

Supplementary Figure 3

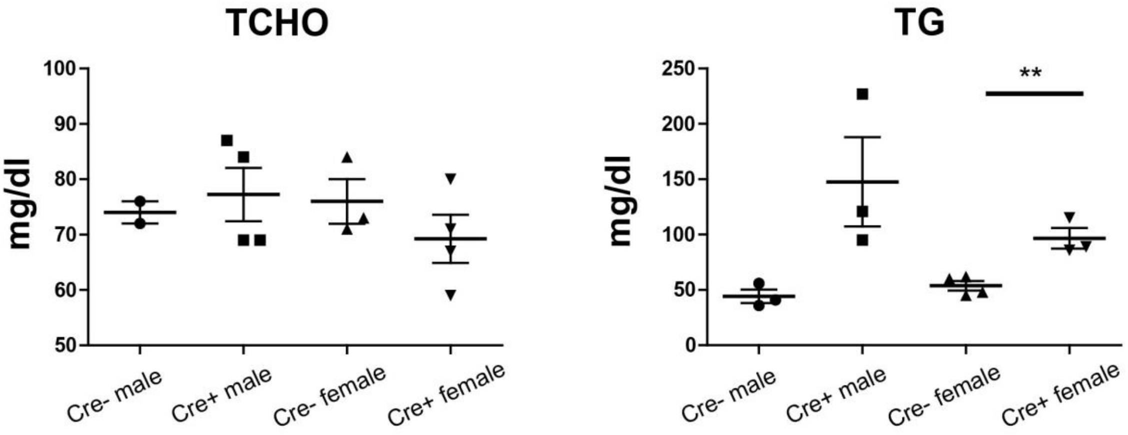

Supplementary Figure 3. Increased plasma triglyceride levels after gonadotrope ablation in females. Total cholesterol (TCHO) and triglyceride (TG) plasma levels in ablated (Cre+) vs control (Cre-) male and female mice. (TCHO: n=2 for Cre- male, n=4 for Cre+ male, n=3 for Cre- female, n=4 for Cre+ female. TG: n=3 for Cre- male, n=3 for Cre+ male, n=4 for Cre- female, n=3 for Cre+ female). ** = Two-tailed t test p-value 0.0055. For statistical details, see Supplementary Data 1. Source data are provided as a Source Data file.

Supplementary Figure 4

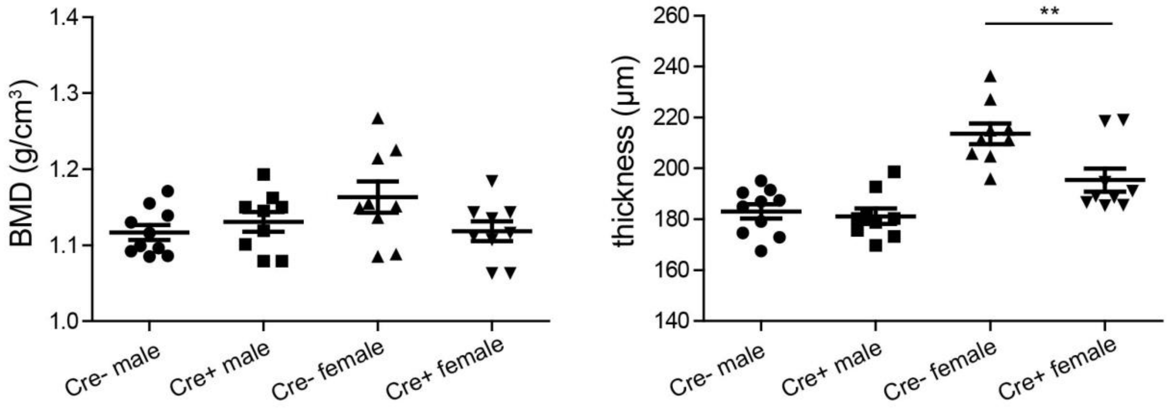

Supplementary Figure 4. Decreased cortical bone thickness of female femurs after gonadotrope ablation. Cortical bone mineral density (BMD) and thickness in gonadotrope-ablated (Cre+) and control (Cre-) male (n=10 Cre- males and n=9 Cre+ males for BMD and thickness) and female mice (n=9 for Cre- females and n=9 for Cre+ females for BMD and thickness). Error bars represent the standard error of the mean. ** = Two-tailed t test p-value 0.0084. For statistical details, see Supplementary Data 1. Source data are provided as a Source Data file.

Supplementary Figure 5

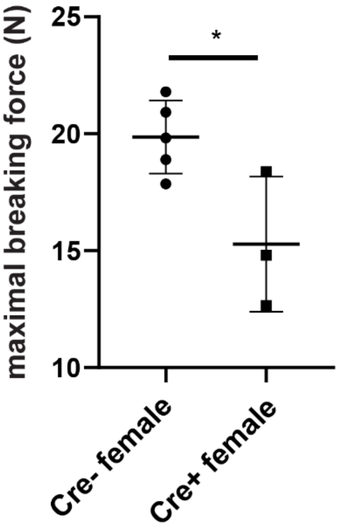

Supplementary Figure 5. Decreased cortical bone strength of female femurs after gonadotrope ablation. Maximal breaking force of femurs from gonadotrope-ablated (Cre+, n=3) and control (Cre-, n=5) female mice. Error bars represent the standard error of the mean. * = P < 0.05. For statistical details, see Supplementary Data 1. Source data are provided as a Source Data file.

Supplementary Figure 6

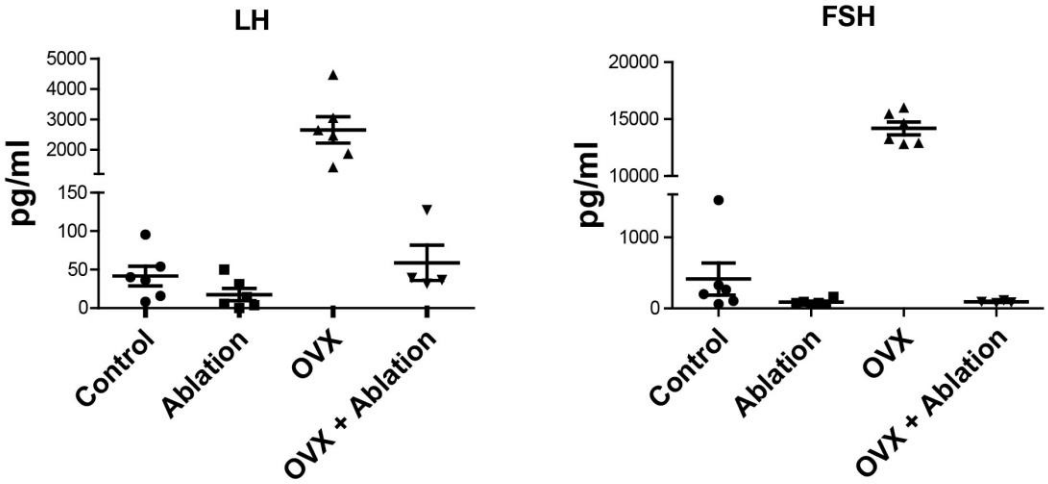

Supplementary Figure 6. Gonadotrope ablation leads to a massive reduction of LH and FSH plasma levels in ovariectomized females. Plasma luteinizing hormone (LH) and follicle-stimulating hormone (FSH), levels in control (n=6 animals), gonadotrope-ablated (ablation, n=6 animals), ovariectomized (OVX, n=6 animals), and gonadotrope-ablated ovariectomized (n=4 animals) female mice. Error bars represent the standard error of the mean. * = P < 0.05. For statistical details, see Supplementary Data 1. Source data are provided as a Source Data file.

Supplementary Figure 7

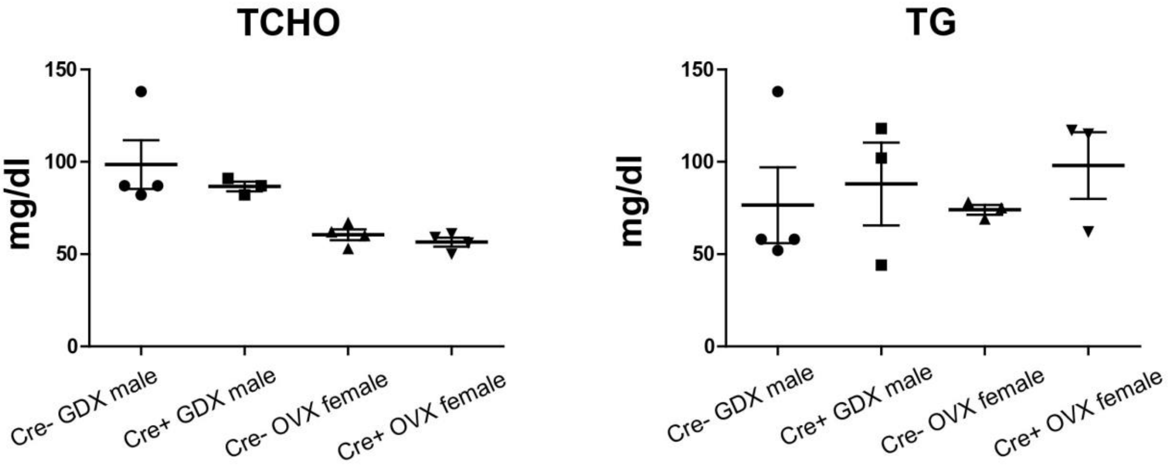

Supplementary Figure 7. Plasma triglyceride and cholesterol levels after gonad removal in combination with gonadotrope ablation. Total cholesterol (TCHO) and triglyceride (TG) plasma levels in gonadotrope-ablated mice lacking gonads (Cre+ OVX and Cre+ GDX) vs control (Cre- OVX and Cre- GDX) male and female mice. (TCHO: n=4 for Cre- GDX male, n=3 for Cre+ GDX male, n=4 for Cre- OVX female, n=4 for Cre+ OVX female. TG: n=3 for Cre- GDX male, n=3 for Cre+ GDX male, n=3 for Cre- OVX female, n=3 for Cre+ OVX female). For statistical details, see Supplementary Data 1. Source data are provided as a Source Data file.

Supplementary Figure 8

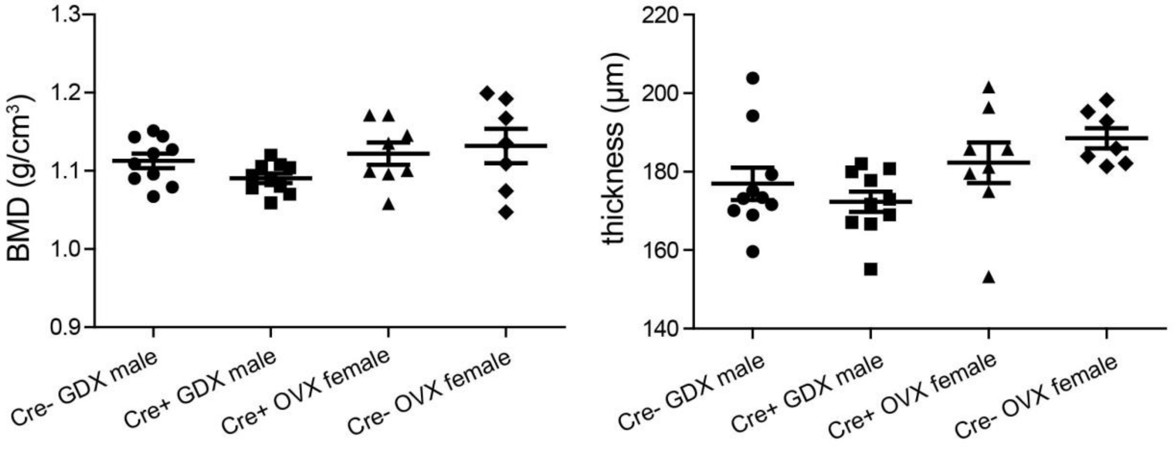

Supplementary Figure 8. Gonadotrope ablation does not affect cortical bone mineral density and thickness in gonadectomized mice. Cortical bone mineral density (BMD) and thickness in gonadotrope-ablated (Cre+) and control (Cre-) gonadectomized (GDX, n=10 Cre- males and n=10 Cre+ males for BMD and thickness) male and ovariectomized (OVX, n=7 Cre- males and n=8 Cre+ males for BMD and thickness) female mice. Error bars represent the standard error of the mean. For statistical details, see Supplementary Data 1. Source data are provided as a Source Data file.

Supplementary Figure 9

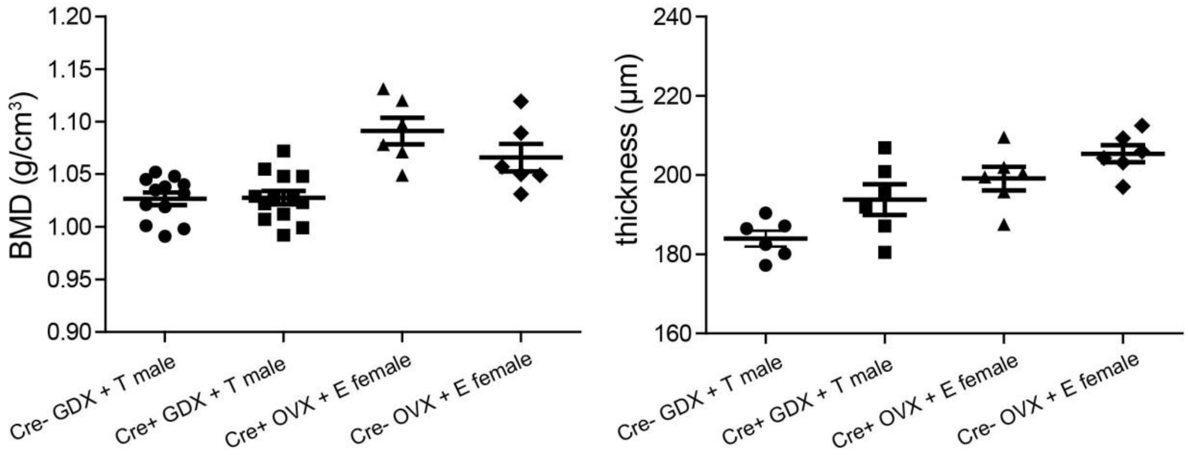

Supplementary Figure 9. Gonadotrope ablation does not affect cortical bone mineral density and thickness in gonadectomized mice with hormone replacement. Cortical bone mineral density (BMD) and thickness in gonadotrope-ablated (Cre+) and control (Cre-) gonadectomized (GDX) male and ovariectomized (OVX) female mice with testosterone (T, n=12 Cre- males for BMD and n=6 Cre- males for thickness and n=13 Cre+ males for BMD and n=6 Cre- and n=6 for Cre+ males for thickness) or estradiol (E, n=6 Cre- females and n=6 Cre- females for BMD and thickness) replacement, respectively. Error bars represent the standard error of the mean. For statistical details, see Supplementary Data 1. Source data are provided as a Source Data file.

Supplementary Figure 10

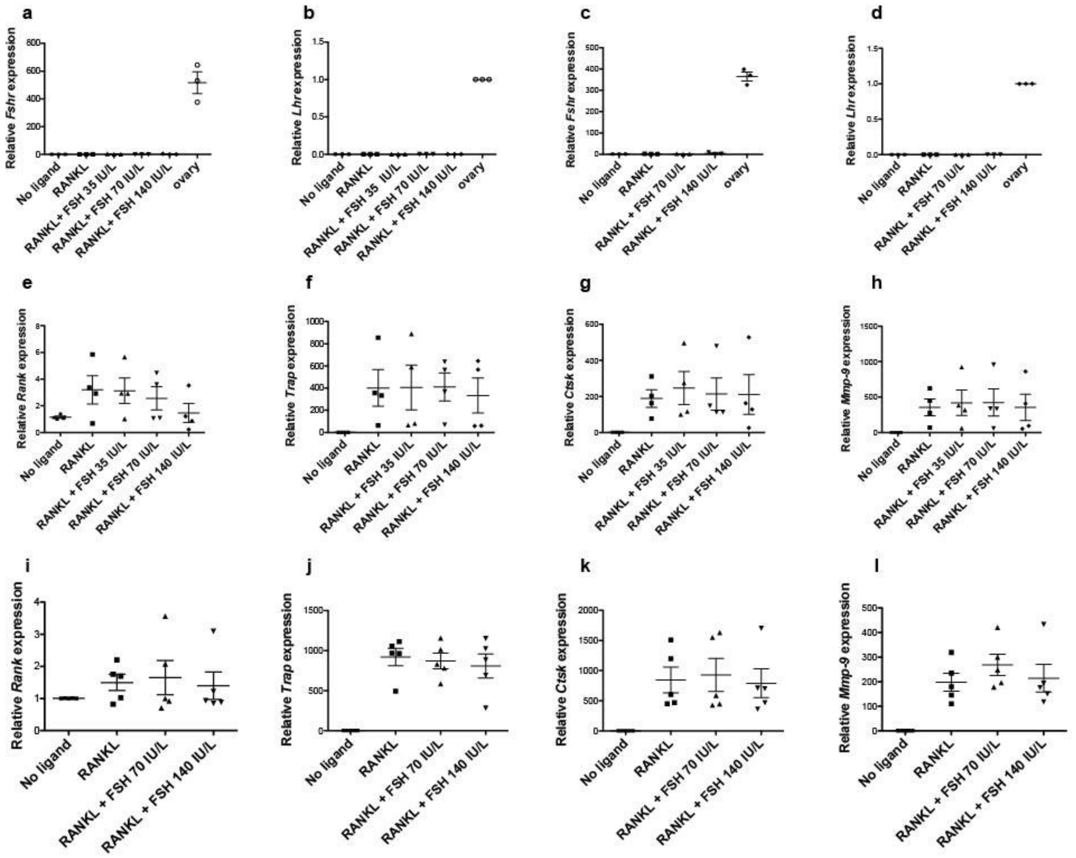

Supplementary Figure 10. FSH does not affect the expression of osteoclast differentiation markers. *Fshr* (**a**) and *Lhcgr* (**b**) expression levels in RAW 264.7 cells in response to the indicated treatments. Expression levels of *Fshr* (**c**) and *Lhcgr* (**d**) in primary murine monocytes under different treatment conditions (note that M-CSF was added in all cases). Ovary RNA was included as a positive control. *Fshr* levels are plotted relative to the no-ligand or M-CSF-only treatment conditions. *Lhcgr* expression levels were plotted relative to ovary as no signal was detected in the cells under any conditions. Expression levels of four osteoclast differentiation markers: receptor activator of nuclear factor κ B (*Rank*) (**e**), tartrate-resistant acid phosphatase (*Trap*) (**f**), cathepsin k (*Ctsk*) (**g**), and matrix metalloproteinase 9 (*Mmp-9*) (**h**) in RAW 264.7 cells in response to the indicated treatments. Expression levels of *Rank* (**i**), *Trap* (**j**), *Ctsk* (**k**), and *Mmp-9* (**l**) in primary murine monocytes in response to the different treatments. Error bars represent the standard error of the mean. For statistical details, see Supplementary Data 1. Source data are provided as a Source Data file.

Supplementary Figure 11

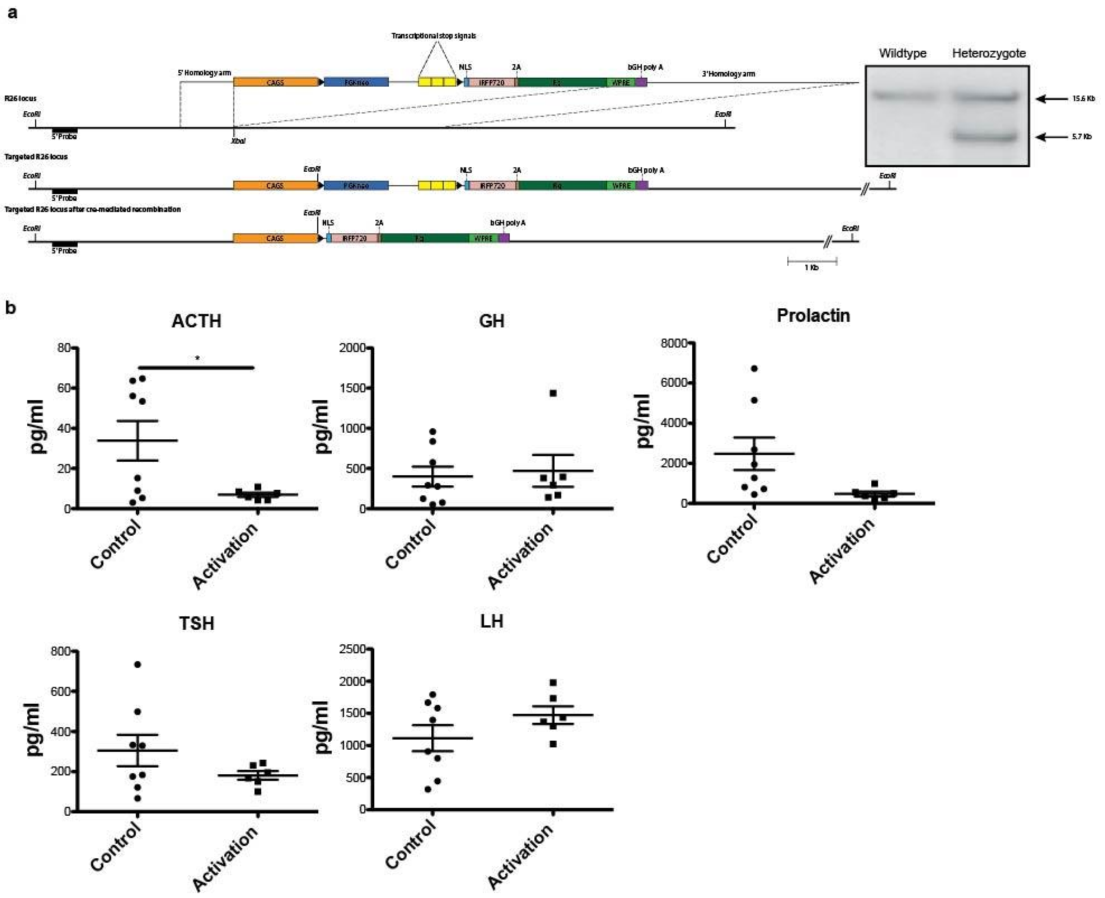

Supplementary Figure 11. Establishment of a gonadotrope activation mouse model. Schematic representation of the targeting strategy used to generate a Cre-dependent DREADD allele in the enhanced *ROSA26* locus. Southern blot analysis of genomic DNA from a wild-type and a heterozygous mutant mouse after digestion with *Eco*RI (**a**). **b** Plasma adrenocorticotropic hormone (ACTH), growth hormone (GH), prolactin, thyroid-stimulating hormone (TSH), and LH from CNO-treated Cre+ (chemogenetically activated) and Cre- (control) ovariectomized (OVX) female mice on high fat diet (HFD). Error bars represent standard error mean. * = $P < 0.05$. For statistical details, see Supplementary Data 1. Source data are provided as a Source Data file.

Supplementary Figure 12

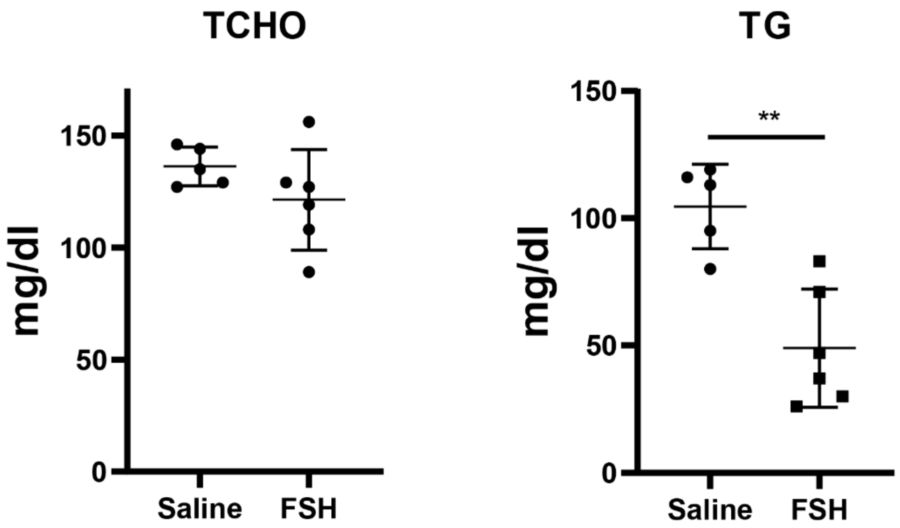

Supplementary Figure 12. Reduced plasma triglyceride levels after FSH treatment. Total cholesterol (TCHO) and triglyceride (TG) levels in FSH (n=6 animals for TCHO and TG)- or saline-treated (n=5 animals for TCHO and TG) OVX/HFD females. Error bars represent standard error mean. ** = $P < 0.01$. For statistical details, see Supplementary Data 1. Source data are provided as a Source Data file.

Supplementary Figure 13

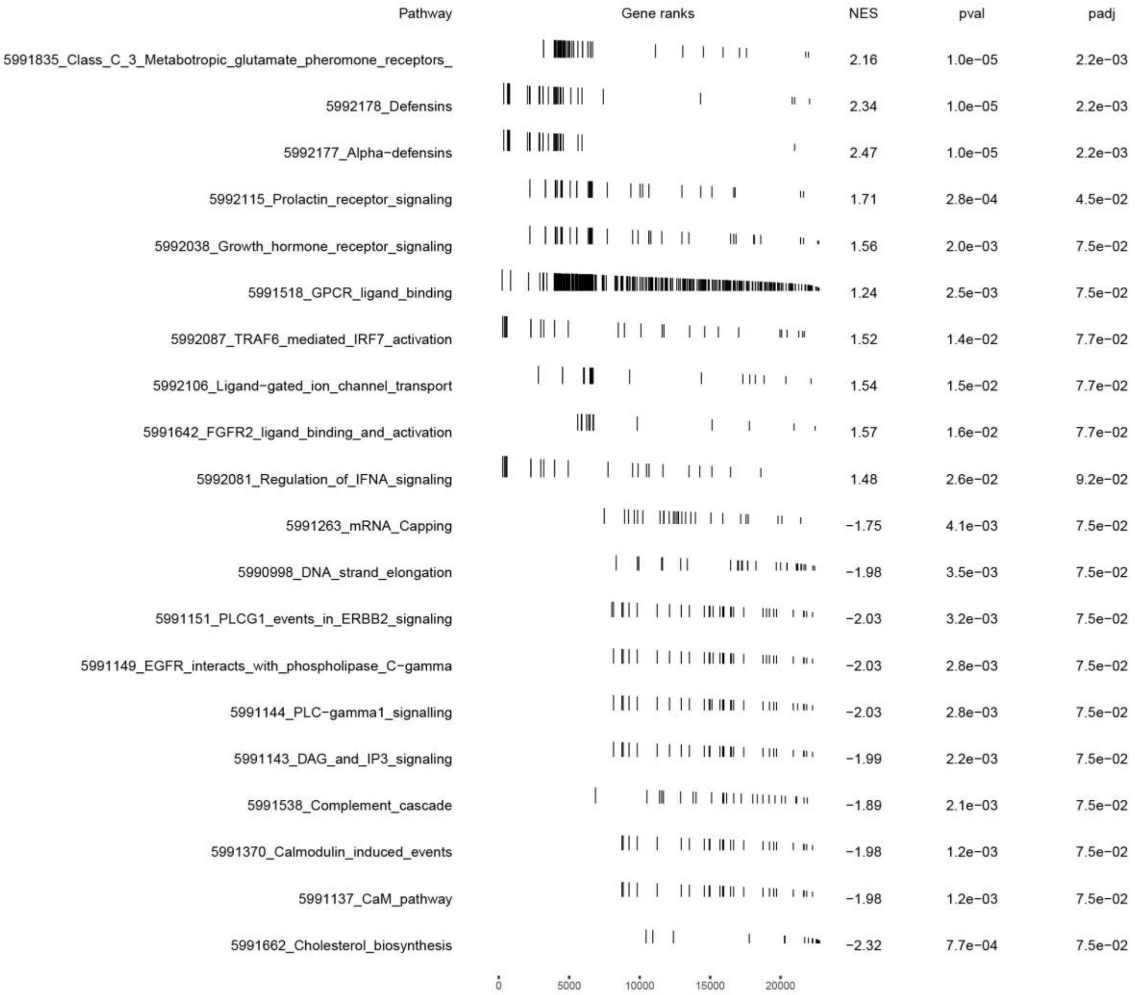

Supplementary Figure 13. Pathway analysis of RNA-seq data after FSH treatment. Gene sets enrichment analysis (GSEA) in transcriptome data of livers prepared from FSH-treated compared to saline treated OVX/HFD females. Source data are provided as a Source Data file.

Supplementary Figure 14

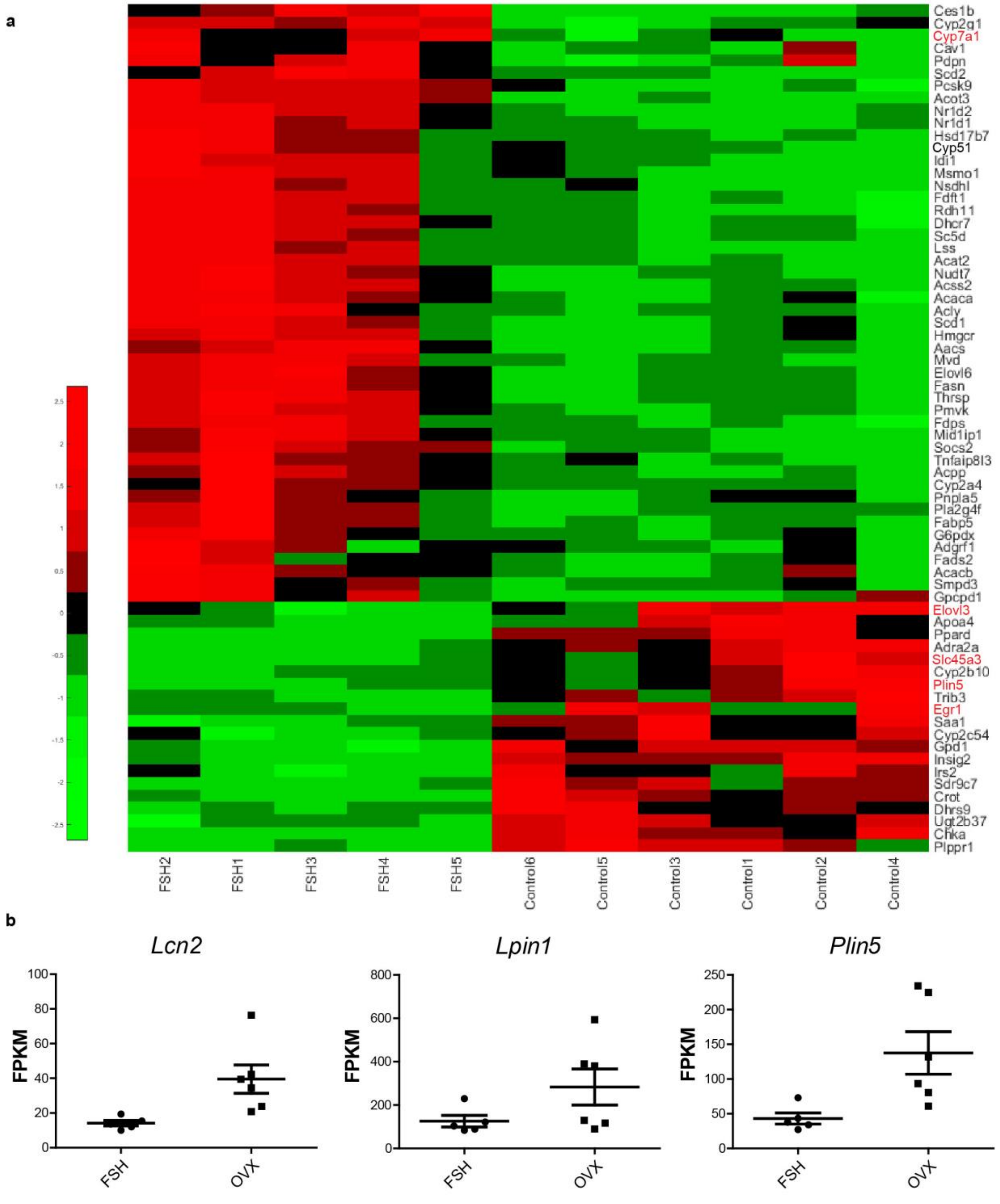

Supplementary Figure 14. Changes in gene expression upon FSH treatment. a Heatmap showing differential gene expression between FSH-treated (FSH/OVX/HFD) and control (OVX/HFD) female mice. Genes marked in red are known to be involved in control of either lipid biosynthetic or catabolic processes. **b** FPKM values for *LCN2* (n=5 animals for FSH treated and n=6 animals for controls), *Lpin1* (n=5 animals for FSH treated and n=6 animals for controls) and *Plin5* (n=5 animals for FSH treated and n=6 animals for controls) in transcriptome data of livers prepared from FSH-treated OVX/HFD females compared to controls (OVX/HFD). Error bars represent standard error mean. For statistical details, see Supplementary Data 1. Source data are provided as a Source Data file.

Supplementary Figure 15

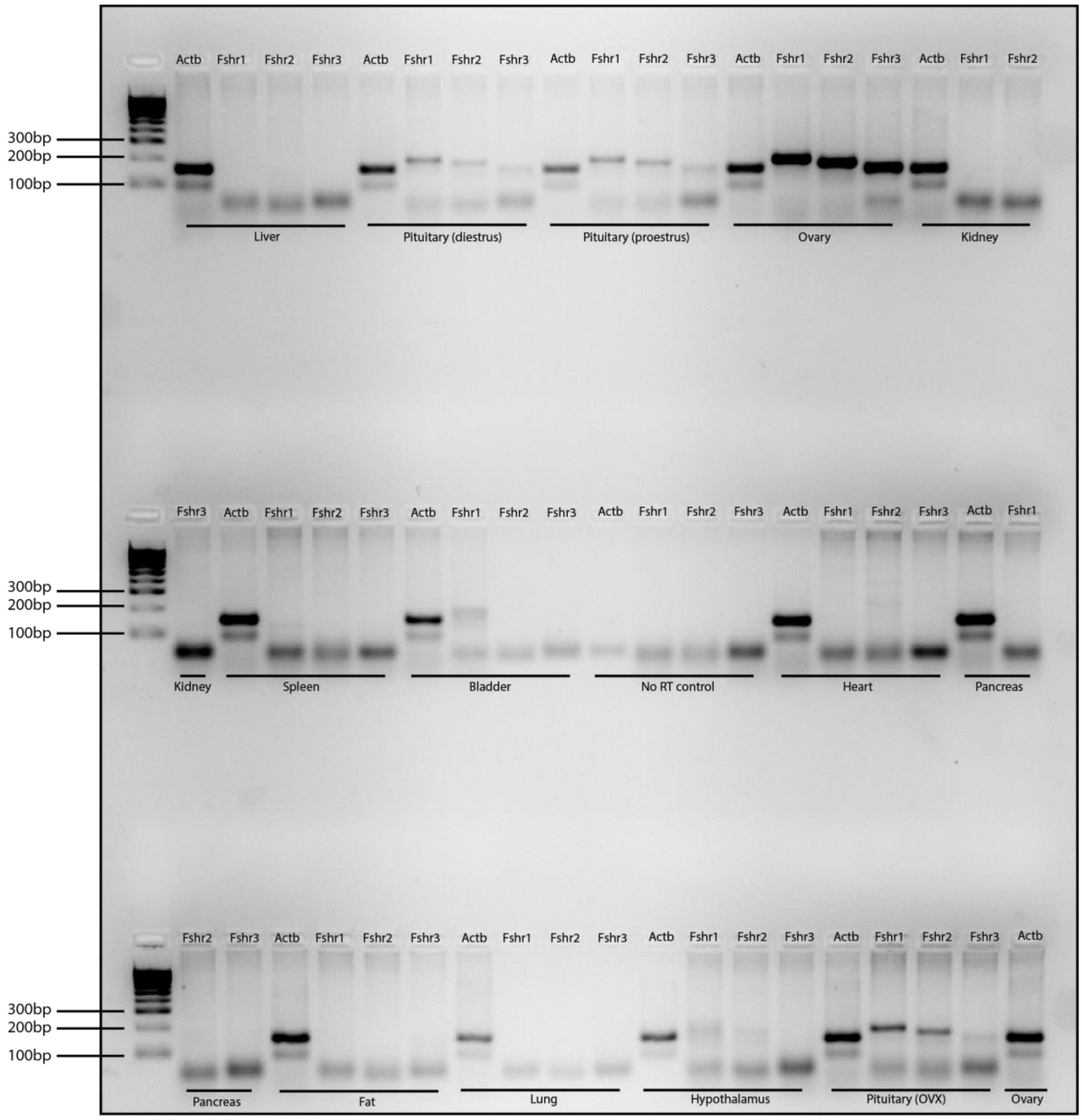

Supplementary Figure 15. Representative RT-PCR analyses of *Fshr* from major organs of adult female mice. Three pairs of primers against *Fshr* were used (Fshr1, Fshr2, Fshr3; see Supplementary Table 1). Shown is a representative gel from one animal. In total n=3 animals were used for liver and pituitary and n=2 animals for the other organs. *Actb*; beta actin was used as a positive control. OVX; ovariectomy.

Supplementary Figure 16

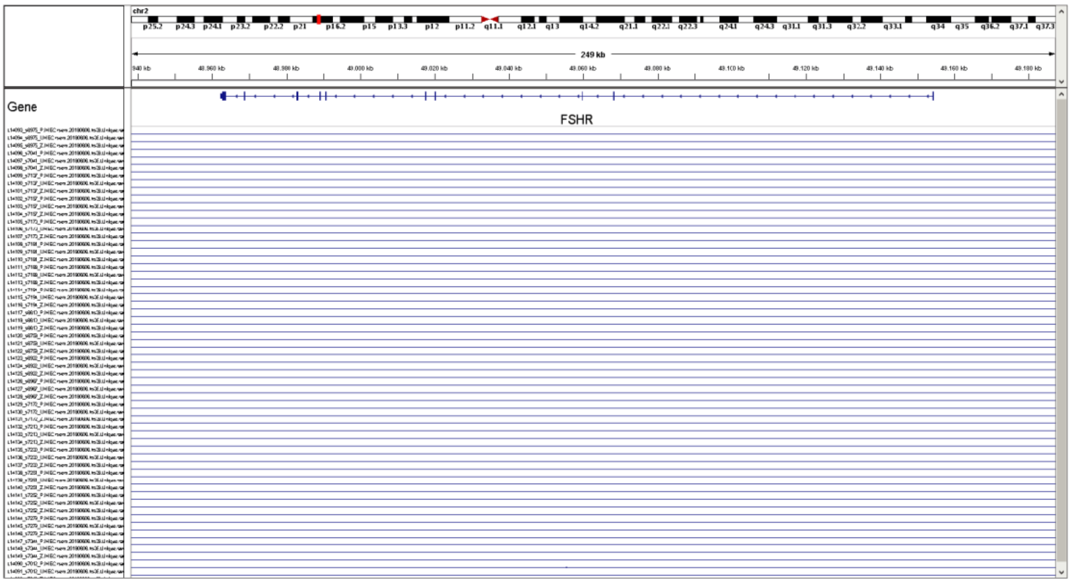

Supplementary Figure 16. Revisiting a published database to identify the expression of FSH receptor in human liver. Detailed genomic view of *FSHR* from RNA-seq data. The IGV browser was used to visualize the genomic loci of *FSHR*. Data were extracted from a published human liver RNA-seq database (GSE105127). In total, 19 donors were used (6 males, 13 females). No reads were mapped in this region.

Supplementary Figure 17

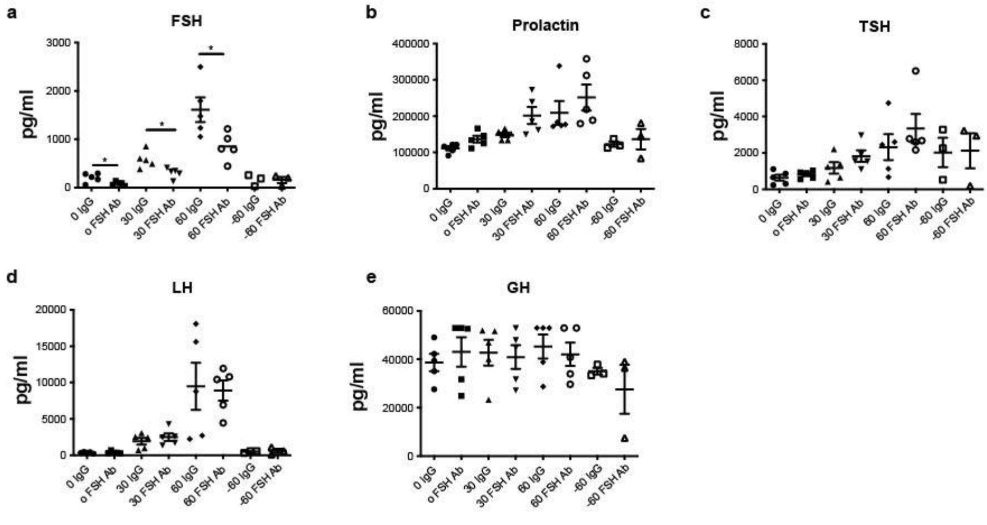

Supplementary Figure 17. *In vitro* pituitary assay analysing effects of FSH antagonism on pituitary hormone secretion. FSH (**a**), prolactin (**b**), TSH (**c**), LH (**d**), and GH (**e**) levels quantified from *in vitro* GnRH-stimulated pituitaries incubated with an anti-FSH antibody or IgG (control), also see experimental paradigm as shown in Fig. 5c. Error bars represent standard error mean. * = P < 0.05. For statistical details, see Supplementary Data 1. Source data are provided as a Source Data file.

Supplementary Figure 18

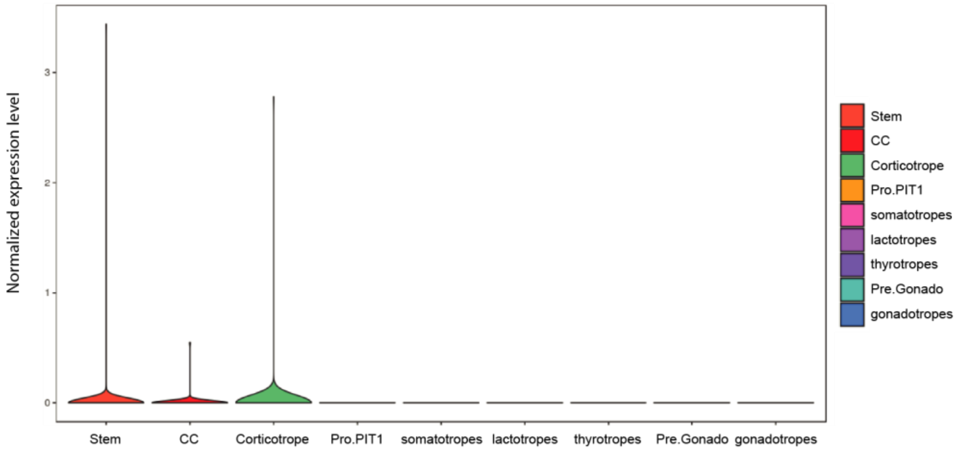

Supplementary Figure 18. Revisiting a published database to identify the expression of FSH receptor in human pituitary. Cell types are shown as follows: stem cells (Stem), cycling cells (CC), corticotropes, progenitors of the PIT-1 lineage (Pro.PIT1), somatotropes, lactotropes, thyrotropes, precursors of gonadotropes (Pre.Gonado) and gonadotropes). Data were extracted from a published single cell RNA-seq database from human pituitary (GSE142653).

Supplementary Figure 19

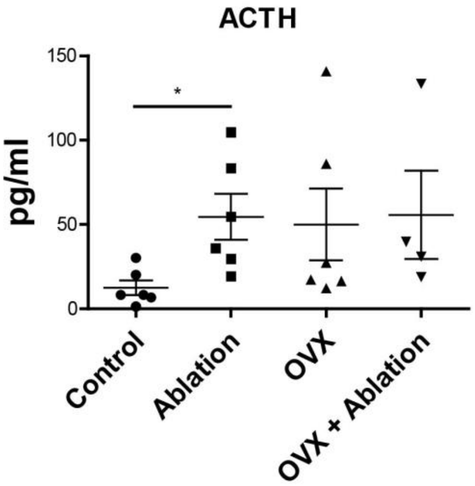

Supplementary Figure 19. Elevated ACTH plasma levels after gonadotrope ablation. Plasma adrenocorticotropic hormone (ACTH) from control, gonadotrope-ablated (ablation), ovariectomized (OVX), and gonadotrope-ablated ovariectomized female mice. Error bars represent the standard error of the mean. * = P < 0.05. For statistical details, see Supplementary Data 1. Source data are provided as a Source Data file.

Supplementary Figure 20

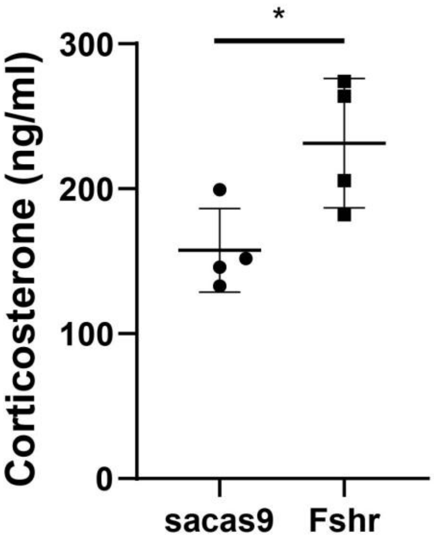

Supplementary Figure 20. Elevated corticosterone levels in pituitary-specific FSHR knock-out females. Plasma corticosterone levels in female mice injected with guide RNA against follicle-stimulating hormone receptor (Fshr) compared to controls. Error bars represent the standard error of the mean. * = $P < 0.05$, For statistical details, see Supplementary Data 1. Source data are provided as a Source Data file.

Supplementary Figure 21

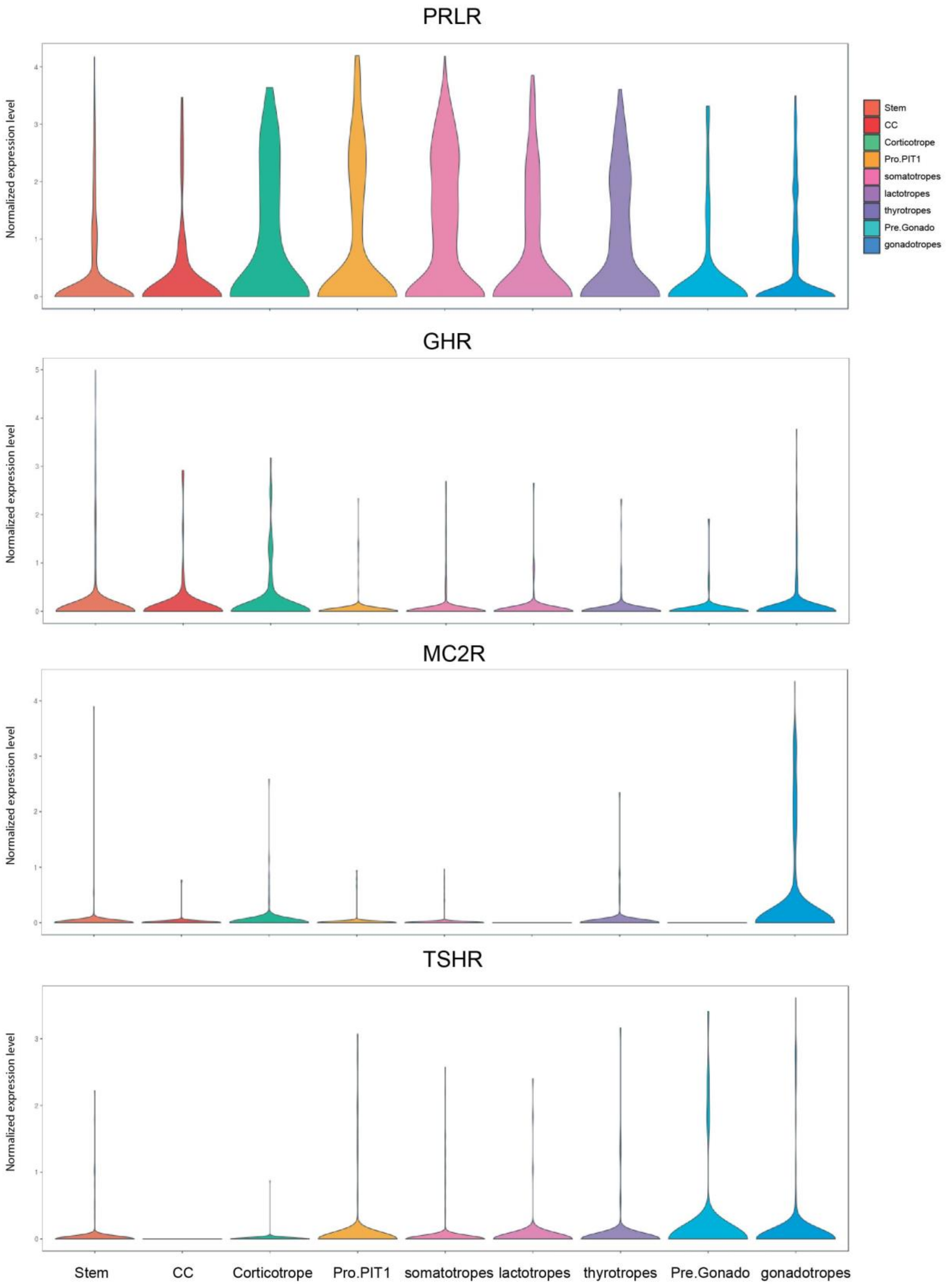

Supplementary Figure 21. Revisiting a published database to identify the expression of hormone receptors in human pituitary. Cell types are shown as follows: stem cells (Stem), cycling cells (CC), corticotropes, progenitors of the PIT-1 lineage (Pro.PIT1), somatotropes, lactotropes, thyrotropes, precursors of gonadotropes (Pre.Gonado) and gonadotropes). Different hormone receptors are shown as follows: prolactin receptor (PRLR), growth hormone receptor (GHR), ACTH receptor (MC2R), thyroid-stimulating hormone receptor (TSHR). Data were extracted from a published single cell RNA-seq database from human pituitary (GSE142653).

Supplementary Figure 22

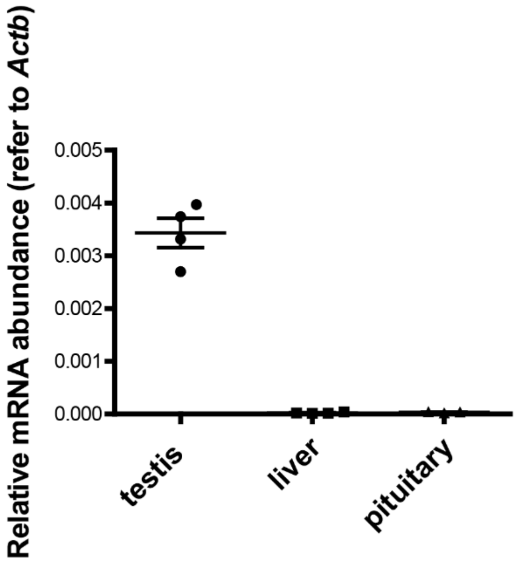

Supplementary Figure 22. The *Fshr* is not expressed in the male pituitary gland. Expression levels of *Fshr* in the testis (n=4 animals), liver (n=4 animals), and pituitary (n=4 animals) from adult male mice were measured using RT-qPCR. Error bars represent standard error mean. For statistical details, see Supplementary Data 1. Source data are provided as a Source Data file.

Supplementary Table 1

**RT-PCR Primers for Supplementary Fig. 15**	**Sequence 5'-3'**
Fshr **F1**	**TGG GCC AGT CGT TTT AGA CA**
Fshr **R1**	**CCT CCA GTT TGC AAA GGC AC**
Fshr **F2**	**GGT CTA TTC CCT GCC CAA CC**
Fshr **R2**	**GTT CAG AGG TTT GCC GCC T**
Fshr **F3**	**ACA ACT GTG CAT TCA ACG GAA CC**
Fshr **R3**	**CAT GGT TGG GCA GGG AAT AGA CC**
Actb **F**	**AAG GAG ATT ACT GCT CTG GCT CCT A**
Actb **R**	**ACT CAT CGT ACT CCT GCT TGC TGA T**
**RT-qPCR Primers for Fig. 5b and Supplementary Fig. 22**	
Fshr **F**	**GGT CTA TTC CCT GCC CAA CC**
Fshr **R**	**GTT CAG AGG TTT GCC GCC T**
Actb **F**	**AAG GAG ATT ACT GCT CTG GCT CCT A**
Actb **R**	**ACT CAT CGT ACT CCT GCT TGC TGA T**
**Primers for CRISPR efficiency verification in Fig. 6b, c**	
Fshr **F**	**GCT ATG ATC CTT GTT CAT CTC TCC**
Fshr **R**	**GAC TCT TGA GAA GGA TAG ACA TCT G**
**RT-qPCR Primers for Supplementary Fig. 10**	
Ctsk F	**CGA AAA GAG CCT AGC GAA CAG A**
**Ctsk R**	**CGC CGA GAG ATT TCA TCC ACC**
Fshr F	**TGT GCT CAC CAA GCT TCG AGT**
Fshr R	**TGC AAG TTG GGT AGG TTG GAG A**
Lhr F	**TGT AAC ACA GGC ATC CGG A**
**Lhr R**	**CGT CCC ATT GAA TGC ATG G**
Mmp-9 F	**CGC TCA TGT ACC CGC TGT AT**
Mmp-9 R	**CCG TGG GAG GTA TAG TGG GA**
Rank F	**TGC AGC TCA ACA AGG ATA CGG**
**Rank F**	**TGA TTC CGT TGT CCC CTG GT**
Rpl19 F	**CGG GAA TCC AAG AAG ATT GA**
**Rpl19 R**	**TTC AGC TTG TGG ATG TGC TC**
Trap F	**GAG GAC GTG TTC TCT GAC CG**
**Trap R**	**GTT GCC ACA CAG CAT CAC TG**

Supplementary Table 1. List of RT-PCR Primers used in this study.

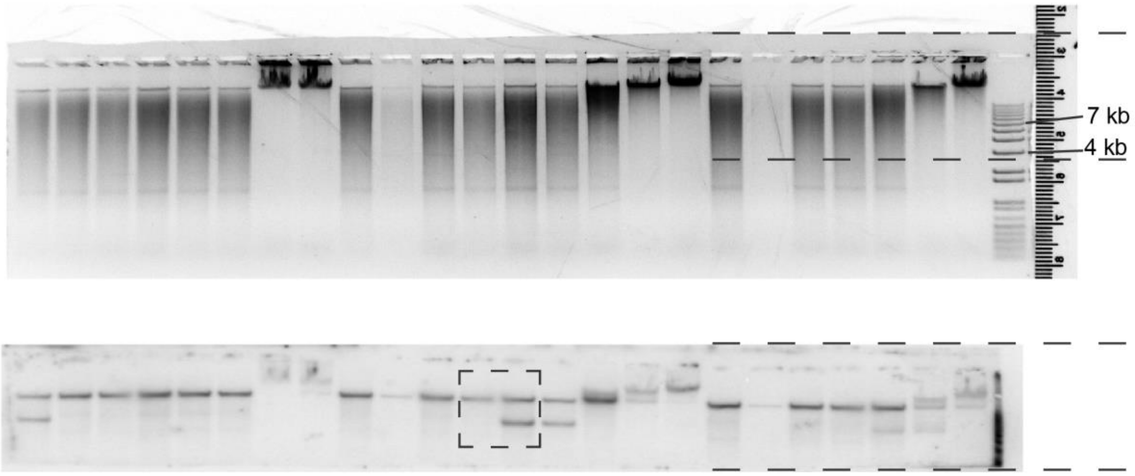

Uncropped Southern Blot from Supplementary Figure 11a. Upper Figure depicts the gel before blotting. Stitched box in the lower blot shows cropped image shown in Supplementary Figure 11a.
