# Supplementary material for: Intra-pituitary follicle-stimulating hormone signaling regulates hepatic lipid metabolism in mice
Source: Nat Commun. 2023 Feb 25;14:1098. doi: 10.1038/s41467-023-36681-z (PMC9968338; doi:10.1038/s41467-023-36681-z)
Supplement: Supplementary file 2 — Description of Additional Supplementary Files [file 41467_2023_36681_MOESM2_ESM.pdf]

Description of Additional Supplementary Files

File Name: Supplementary Data 1
Description: Statistical information for all experiments.
